# PI3K and Inhibitor of Apoptosis Proteins Modulate Gentamicin- Induced Hair Cell Death in the Zebrafish Lateral Line

**DOI:** 10.3389/fncel.2017.00326

**Published:** 2017-10-18

**Authors:** Heather Wiedenhoft, Lauren Hayashi, Allison B. Coffin

**Affiliations:** ^1^College of Arts and Sciences, Washington State University, Vancouver, WA, United States; ^2^Knight Cancer Institute, Oregon Health & Science University, Portland, OR, United States; ^3^Department of Integrative Physiology and Neuroscience, Washington State University, Vancouver, WA, United States

**Keywords:** zebrafish, hair cell protection, hearing loss, aminoglycosides, lateral line, ototoxicity, programmed cell death, strain difference

## Abstract

Inner ear hair cell death leads to sensorineural hearing loss and can be a direct consequence of aminoglycoside antibiotic treatment. Aminoglycosides such as gentamicin are effective therapy for serious Gram-negative bacterial infections such as some forms of meningitis, pneumonia, and sepsis. Aminoglycosides enter hair cells through mechanotransduction channels at the apical end of hair bundles and initiate intrinsic cell death cascades, but the precise cell signaling that leads to hair cell death is incompletely understood. Here, we examine the cell death pathways involved in aminoglycoside damage using the zebrafish (*Danio rerio*). The zebrafish lateral line contains hair cell-bearing organs called neuromasts that are homologous to hair cells of the mammalian inner ear and represents an excellent model to study ototoxicity. Based on previous research demonstrating a role for p53, Bcl2 signaling, autophagy, and proteasomal degradation in aminoglycoside-damaged hair cells, we used the Cytoscape GeneMANIA Database to identify additional proteins that might play a role in neomycin or gentamicin ototoxicity. Our bioinformatics analysis identified the pro-survival proteins phosphoinositide-dependent kinase-1 (PDK1) and X-linked inhibitor of apoptosis protein (Xiap) as potential mediators of gentamicin-induced hair cell damage. Pharmacological inhibition of PDK1 or its downstream mediator protein kinase C facilitated gentamicin toxicity, as did *Xiap* mutation, suggesting that both PI3K and endogenous Xiap confer protection. Surprisingly, aminoglycoside-induced hair cell death was highly attenuated in wild type Tupfel long-fin (TL fish; the background strain for the *Xiap* mutant line) compared to wild type ^∗^AB zebrafish. Pharmacologic manipulation of p53 suggested that the strain difference might result from decreased p53 in TL hair cells, allowing for increased hair cell survival. Overall, our studies identified additional steps in the cell death cascade triggered by aminoglycoside damage, suggesting possible drug targets to combat hearing loss resulting from aminoglycoside exposure.

## Introduction

Aminoglycosides antibiotics, including neomycin and gentamicin, cause hearing loss and vestibular dysfunction in 20–30% of patients who take these drugs (Rizzi and Hirose, 2007; Xie et al., 2011; Schacht et al., 2012). However, these antibiotics are still frequently used world-wide because of their relatively low cost and high efficacy in fighting devastating infectious diseases such as tuberculosis, sepsis, and other severe Gram-negative bacterial infections (Maurin and Raoult, 2001; Rizzi and Hirose, 2007). Aminoglycosides cause hearing loss by killing sensory hair cells in the inner ear, which eliminates the first neural step in transforming acoustic energy into neural impulses (Schacht et al., 2012). Damage to mammalian hair cells is permanent, as they do not regenerate. It is therefore critical that we understand how aminoglycosides cause hair cell death so that we may develop targeted therapies to intervene in the damage process and preserve hearing.

Cell death signaling is traditionally broken into two domains: apoptosis and necrosis. Apoptosis is classified morphologically by nuclear condensation and biochemically by caspase activation, while necrosis is distinct, with nuclear and plasma membrane swelling (Zimmermann et al., 2001; Danial and Korsmeyer, 2004). However, this binary classification fails to fully encompass the complexity of cell death signaling that occurs in many tissues, including the ear (Forge and Schacht, 2000; Matsui et al., 2002; Jiang et al., 2006a; Coffin et al., 2013b). Studies in the past decade demonstrate that aminoglycosides activate a complex set of signaling cascades in damaged hair cells, and that not all hair cells respond to aminoglycosides in the same biochemical manner (Jiang et al., 2006a; Taylor et al., 2008; Coffin et al., 2013b). For example, *in vitro* and *in vivo* studies in chickens and rodents suggest that classical apoptosis plays a dominant role in aminoglycoside damage, primarily activating the mitochondrial cell death pathway driven by caspase-9 and caspase-3 (e.g., Forge and Li, 2000; Cunningham et al., 2002; Matsui et al., 2002, 2004; Cheng et al., 2003). However, other research in mammals and zebrafish demonstrates caspase-independent cell death *in vivo*, albeit with involvement of mitochondrial proteins (Jiang et al., 2006a; Owens et al., 2007; Tabuchi et al., 2007; Coffin et al., 2013a,b). These contradictory data likely result from differences in species and experimental conditions, including *in vitro* vs. *in vivo* differences and differences in drug treatment paradigms. Reactive oxygen species formation is a hallmark feature in many aminoglycoside ototoxicity studies, and antioxidants confer some level of protection (Hirose et al., 1999; McFadden et al., 2003; Choung et al., 2009; Poirrier et al., 2010; Esterberg et al., 2016). Other studies suggest involvement of numerous cell death and survival cascades, including c-Jun N-terminal kinase (JNK) and p53 signaling (Wang et al., 2003; Sugahara et al., 2006; Coffin et al., 2013a; Anttonen et al., 2016). Despite these studies, we still have an incomplete picture of the signaling events that occur in aminoglycoside-damaged hair cells. A better understanding of cell death and survival signaling due to aminoglycoside exposure will provide more targets for therapeutic intervention.

The present study uses the larval zebrafish lateral line to better understand cell death processes after aminoglycoside exposure. The lateral line is used by zebrafish to detect near field vibrations in the water caused by abiotic or biotic sources such as prey, predators, or water current (Montgomery et al., 1997; Coombs et al., 2014). The lateral line system contains clusters of neuromasts—sensory hair and supporting cells encapsulated in a jelly-like cupula—that are arranged along the head and trunk of the fish. Lateral line hair cells are structurally and functionally similar to the hair cells of the mammalian inner ear and show similar responses to aminoglycosides and other hair cell toxins (Harris et al., 2003; Ou et al., 2007; Coffin et al., 2010). In the lateral line, neomycin and gentamicin activate distinct, yet somewhat overlapping, responses in damaged hair cells, suggesting that not all cell death responses are common across aminoglycosides and that a greater understanding of these differences is necessary to develop appropriate therapeutics (Coffin et al., 2009, 2013a,b; Owens et al., 2009; Hailey et al., 2017). Neomycin induces changes in calcium mobilization, mitochondrial membrane potential, and reactive oxygen species generation, and damage is dependent on the mitochondrial protein Bax (Owens et al., 2007; Coffin et al., 2013a; Esterberg et al., 2013, 2014, 2016). Although gentamicin toxicity in the lateral line is less well-studied, prior research shows that gentamicin-induced damage is independent of Bax and substantially dependent on p53 signaling (Coffin et al., 2013a).

In a previous study, we screened a cell death inhibitor library to identify novel regulators of aminoglycoside-induced hair cell death in the lateral line (Coffin et al., 2013b). This study identified several compounds that modulate aminoglycoside-induced hair cell death in the lateral line, including a Bax channel blocker, the p53 inhibitor pifithrin-α (PFTα), the Omi/HtrA2 inhibitor Ucf-101, and the autophagy inhibitor 3-MA (Coffin et al., 2013a,b). Here, we used this cell death inhibitor dataset as the input for pathway analysis using Cytoscape GeneMANIA to identify additional protein targets that may modulate aminoglycoside ototoxicity. We generated a list of molecular targets for each pharmacological reagent from the inhibitor dataset, basing our target selection on the literature demonstrating specific targets for each inhibitor. Our list contains 36 genes that our previous work suggests may modulate aminoglycoside ototoxicity, with some gene products implicated in neomycin toxicity, some in gentamicin toxicity, and some in response to either aminoglycoside. Pathway analysis yielded a complex network of potentially interacting signaling molecules in response to neomycin or gentamicin application.

We then used pharmacological or genetic manipulation to examine how these protein targets influence aminoglycoside-induced hair cell death. We selected two candidate classes of molecules: phosphoinositide-dependent kinase-1 (PDK1) and inhibitor of apoptosis proteins (IAPs), for further analysis, based on the potentially central role of these molecules, the paucity of information about these molecules in the auditory periphery, and the availability of pharmacological and genetic reagents for experimental use. We elected to focus on modulators of gentamicin damage because there is relatively little information on gentamicin ototoxicity in the lateral line, while neomycin damage is more understood (e.g., Harris et al., 2003; Esterberg et al., 2013, 2014, 2016). We found that PDK1 plays a protective role in gentamicin-damaged hair cells, as does X-linked inhibitor of apoptosis protein (Xiap). The *Xiap* mutant fish were created on a Tupfel long-fin (TL) background, and through comparisons with other wild type fish lines we discovered that hair cells in TL fish are less sensitive to aminoglycoside damage than hair cells in ^∗^AB fish, likely owing to differences in p53.

## Materials and Methods

### Pathway Analysis

Based on our previous work (Coffin et al., 2013a,b), we generated a list of 36 genes that may modulate neomycin or gentamicin toxicity in the lateral line. Accession numbers and nucleotides for each gene were collected from the NCBI database (Brown et al., 2015). We used this gene list as input for pathway analysis of hair cell responses to neomycin or gentamicin damage. Supplementary Table 1 contains the list of input genes. Functional annotation of genes was performed using The Database for Annotation, visualization and Integrated Discovery (DAVID) Gene-Enrichment and Functional Annotation Analysis (Dennis et al., 2003; Huang et al., 2007; Wilhelm et al., 2012). The default EASE threshold of 0.1.0 was used. Global co-expression networks were then constructed using the Cytoscape app GeneMANIA (Campanaro et al., 2007; Montojo et al., 2010; Vied et al., 2014; Hausen et al., 2017). The desired organism used was human, with the options to display up to 50 related genes, 20 related attributes, and automatic weighting. Interactions were designated as genetic interactions, shared protein domains, pathway, co-expression, physical interactions, and predicted interactions. These different interactions are represented by different colored lines in the final output. From this analysis, we selected candidate proteins for pharmacological or genetic manipulation during aminoglycoside exposure in larval zebrafish.

### Animals

Experiments were conducted on ^∗^AB wild type fish unless specifically noted. The *Xiap^sa2739^* mutant fish, which have a premature stop codon in the *Xiap* gene, were created by the Sanger Institute on a TL background (zfin.org). *Xiap* mutants were purchased through the Zebrafish International Resource Center (ZIRC) and bred as a homozygous line. Wild type TL fish were also purchased through ZIRC. All fish were reared in the zebrafish facility at Washington State University, Vancouver. Larvae were produced through group matings and raised at 28°C in Petri dishes containing fish water. All experiments were performed in E2 embryo medium (EM: 1 mM MgSO_4_, 0.15 mM KH_2_PO_4_, 1 mM CaCl_2_, 0.5 mM KCl, 15 mM NaCl, 0.05 mM Na_2_HPO, and 0.7 mM NaHCO_3_ in dH_2_O, pH 7.2; Westerfield, 2000). Animals were used at 5–6 days post-fertilization (dpf) because at this age their hair cells exhibit mature sensitivity to known hair cell toxins (Murakami et al., 2003; Santos et al., 2006; Ou et al., 2007). This study was carried out in accordance with the recommendations of the American Veterinary Medical Association and the Institutional Animal Care and Use Committee at Washington State University. The protocol was approved by the Institutional Animal Care and Use Committee at Washington State University.

### Drug Exposure

Stock solutions of the aminoglycosides gentamicin and neomycin (Sigma-Aldrich, St. Louis, MO, United States) were diluted in EM to final concentrations of 25–400 μM. The ototoxic chemotherapy agent cisplatin was also used for one set of experiments. Cisplatin (MWI Animal Health, Boise, ID, United States) was diluted in EM to final concentrations of 250–1000 μM. For all experiments, fish were divided into treatment groups of 6–20 fish per group and placed in custom transfer baskets in a six-well plate. In order to understand the differences between neomycin- and gentamicin-induced hair cell death, we used either an acute treatment paradigm (30 min drug exposure, 60 min recovery in EM), or a continuous treatment paradigm (6 h drug exposure), as previous research demonstrates that these treatments activate distinct sets of cell death events (Coffin et al., 2009, 2013a,b; Owens et al., 2009). The acute exposure paradigm was used for neomycin treatment, while the continuous exposure paradigm was used for gentamicin.

In order to examine cell death, we used the following cell signaling modulators: PHT-427, which is a dual inhibitor of Akt and PDK1 proteins (Selleck Chemicals, Houston, TX, United States), LCL-161, a Smac/Diablo mimetic that acts as an antagonist of IAPs (Selleck Chemicals), an Akt inhibitor (Akt Inhibitor VIII, EMD Millipore, Billerica, MA, United States), the PDK1 inhibitor BX795 (UBPBio, Aurora, CO, United States), the protein kinase C (PKC) inhibitor Calphostin C (EMD Millipore), the p53 inhibitor PFTα, or the Mdm2 inhibitor nutlin-3a, which stabilizes p53. PFTα and nutlin-3a were purchased from Sigma. Cell death modulators were added 1 h prior to drug treatment and remained present during aminoglycoside exposure. Cell death modulators were reconstituted in DMSO. Control fish were given a corresponding amount of DMSO, ranging from 0.001 to 1% total volume to match the DMSO concentrations in the inhibitor treatment.

### Hair Cell Assessment

To quantify hair cell survival, fish were treated with the vital dye 2-(4-(dimethylamino)styryl)-*N*-ethylpyridinium iodide (DASPEI, Life Technologies, Grand Island, NY, United States). Fish were incubated in 0.005% DASPEI for 15 min, then rinsed twice in fresh EM and anesthetized with 0.001% buffered 3-aminobenzoic acid ethyl ester methanesulfonate (MS-222, Argent Labs, Redmond, WA, United States) prior to imaging with a Leica M165FC fluorescent stereomicroscope (Leica Microsystems, Buffalo Grove, IL, United States). Ten pre-determined anterior neuromasts (IO1, IO2, IO3, M2, IO4, O2, MI1, MI2, SO1, and SO2; Raible and Kruse, 2000) per fish were then assigned a score from 0 to 2 based on brightness of DASPEI labeling, with 0 being no visible neuromast, 1 indicating visible but faint labeling, and 2 indicating bright labeling (Harris et al., 2003; Coffin et al., 2009; Owens et al., 2009). Aggregate scores were given per fish as a sum of its 10 neuromast scores (score of 0–20 per fish), and data were normalized to the control group for each experiment.

For a sub-set of experiments, direct hair cells counts were used to validate DASPEI scores (Coffin et al., 2009, 2013a). Fish were euthanized with 0.002% buffered MS-222 and fixed in 4% paraformaldehyde overnight at 4°C. Fish were then rinsed in phosphate-buffered saline (PBS, Life Technologies) and blocked in PBS supplemented with 5% normal goat serum and 0.1% Triton-X (both from Sigma-Aldrich). Fish were then incubated overnight at 4°C in anti-parvalbumin, a specific hair cell label, in PBS with 0.1% Triton-X and 1% goat serum (1:500 antibody dilution, EMD Millipore). Fish were rinsed in fresh PBS + Triton-X, incubated in secondary antibody (Alexa Fluor goat anti-mouse 488 or 568, Life Technologies) for 4 h at room temperature, rinsed again, and stored at 4°C in a 1:1 solution of PBS:glycerol. The fish were then mounted on bridged coverslips and viewed using a Leica DMRB compound fluorescent microscope at 40× magnification, or with a Leica SP8 scanning confocal microscope. Hair cells from the same five neuromasts situated under the eye (IO1, IO2, IO3, M2, and OP1; Raible and Kruse, 2000) were counted and summed to arrive at one value per fish.

### UV-Induced Cell Death

In order to examine cell death in other cells, we used UV light exposure, which causes DNA damage and cell death in a variety of cell types (Zeng et al., 2009; Yabu et al., 2015; Mao et al., 2017). Three dpf larvae (^∗^AB or TL) were exposed to UV light for 90 s, then allowed to recover in either EM or PFTα for 3 h. Fish were then labeled with 5 μg/ml acridine orange (AO) for 45 min (Life Technologies; Kong et al., 2016), rinsed twice in fresh EM, anesthetized with MS-222, and imaged on a Leica SP8 confocal microscope. Identical laser, gain, and offset setting were used for all images. AO-labeled cells were quantified from a 10,000 μm^2^ region of the ventral surface of the head, where the gill arches meet.

### Data Analysis

Data were analyzed by one- or two-way ANOVA (as appropriate) using GraphPad Prism version 7. All data are presented as mean ± 1 SD. With power analysis using 10 animals per group, our standard sample size, we can detect a difference of 12% with 95% power, with type I and type II errors of 0.05. For experiments with *N* = 6, this allows us to detect a 15% difference.

## Results

### PI3K or IAP Inhibition Modulates Hair Cell Death

Pathway analysis yielded a complex network of potentially interacting signaling molecules in response to neomycin or gentamicin application (Supplementary Figure 1 and Data Sheets 1, 2). Here, we focus on PDK1 and IAP as potential modulators of hair cell death, as both molecules appeared on one or both pathway diagrams (for neomycin and gentamicin).

**FIGURE 1:**
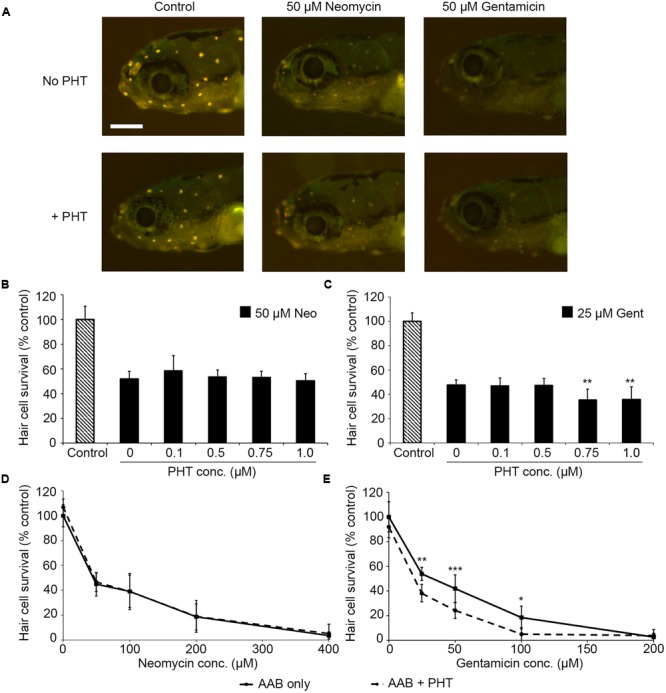
The PDK1/Akt inhibitor PHT-427 sensitizes hair cells to gentamicin damage but not to neomycin. **(A)** Representative images showing DASPEI-labeled fish from the indicated groups. Scale bar = 200 μm and applies to all panels. **(B)** PHT dose–response function with 50 μM neomycin. PHT does not alter neomycin sensitivity (one-way ANOVA, *F*_4,50_ = 1.68, *p* = 0.17). **(C)** PHT increases sensitivity to gentamicin toxicity (one-way ANOVA, *F*_4,43_ = 6.94, *p* < 0.001). Bonferroni-corrected *post hoc* analysis demonstrates significant sensitization at 0.75 or 1 μM PHT. **(D)** 0.75 μM PHT does not alter the neomycin dose–response function (two-way ANOVA, *F*_1,86_ = 1.04, *p* = 31). **(E)** 0.75 μM PHT significantly sensitizes hair cells to gentamicin (two-way ANOVA with inhibitor presence as the factor, *F*_1,67_ = 31.95, *p* < 0.001). In panels **(D,E)**, solid lines are aminoglycoside (AAB) only, dashed lines are aminoglycoside with 0.75 μM PHT. Bonferroni-corrected *post hoc* analysis shows significant differences at 25, 50, and 100 μM gentamicin. ^∗^*p* < 0.05, ^∗∗^*p* < 0.01, ^∗∗∗^*p* < 0.001. *N* = 6–12 fish/treatment.

**FIGURE 2:**
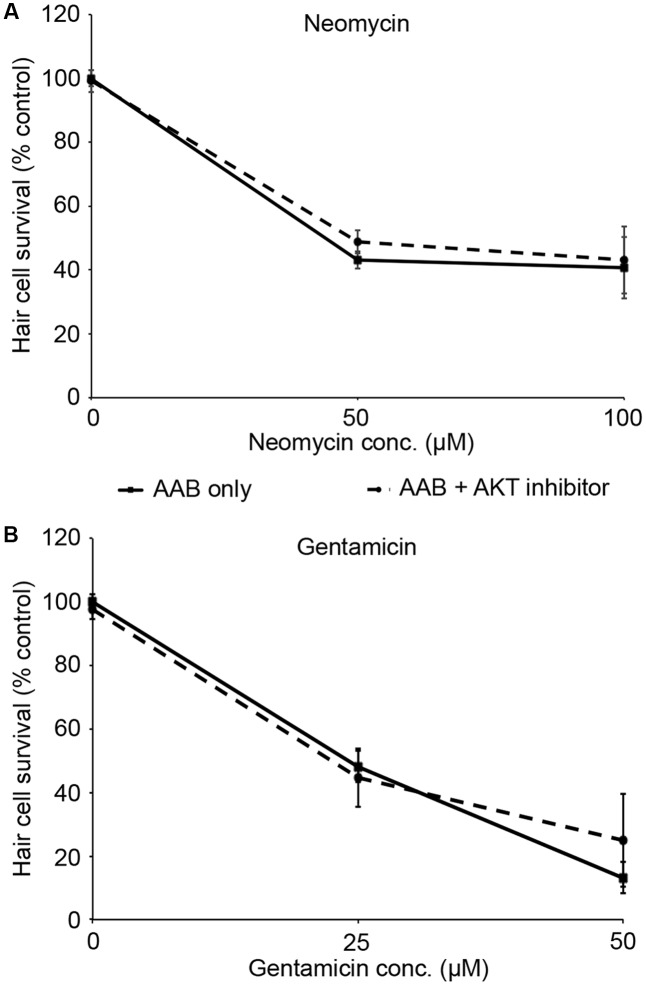
Akt inhibition does not modulate aminoglycoside-induced hair cell death. Fish were treated with 1 μM Akt inhibitor and variable concentrations of neomycin **(A)** or gentamicin **(B)**. Solid lines are aminoglycoside (AAB) only, dashed lines are aminoglycoside with Akt inhibitor. Data were analyzed by two-way ANOVA with inhibitor presence as the factor. *F*_1,55_ = 2.31, *p* = 0.13 (neomycin), *F*_1,59_ = 0.89, *p* = 0.35 (gentamicin). *N* = 10–12 fish/treatment.

**FIGURE 3:**
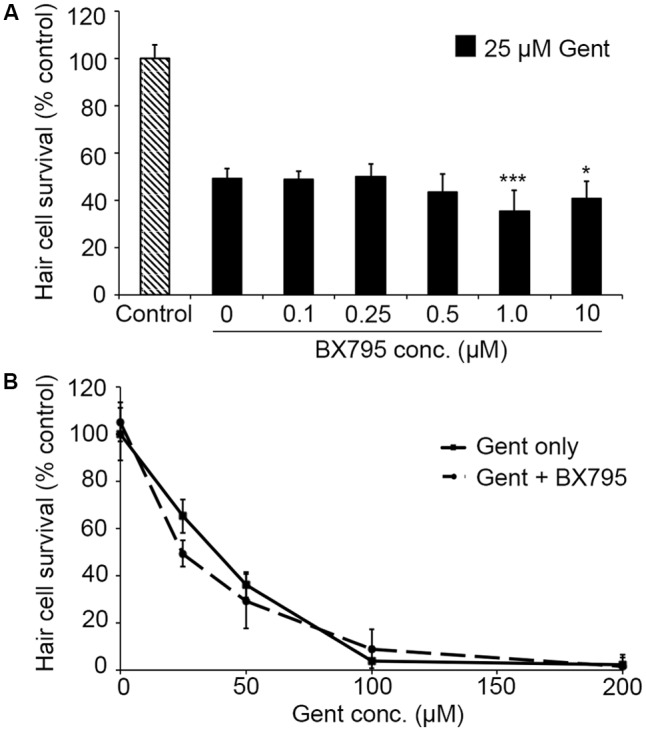
The PDK1 inhibitor BX795 sensitizes hair cells to gentamicin damage. **(A)** BX795 exhibits dose-dependent sensitization (one-way ANOVA, *F*_5,60_ = 8.807, *p* < 0.001). Significant sensitization is seen with 1 or 10 μM BX795 against 25 μM gentamicin (^∗∗∗^*p* < 0.001 and ^∗^*p* < 0.05, respectively).**(B)** A trend toward BX795 sensitization is seen across the gentamicin dose–response function, although this effect is not significant (two-way ANOVA with inhibitor presence as the factor, *F*_1,101_ = 3.501, *p* = 0.06). Solid lines are gentamicin only, dashed lines are gentamicin with BX795. *N* = 8–13 fish/treatment.

PHT-427, a dual inhibitor of PDK1 and Akt, sensitized hair cells to gentamicin damage, with 750 nM PHT conferring significant sensitization (**Figure 1**; one-way ANOVA, *p* < 0.001). 750 nM PHT increased gentamicin-induced hair cell damage by 13–17%, depending on the gentamicin concentration (**Figure 1E**). There was no sensitization observed with 200 μM gentamicin, as this concentration damaged all hair cells without PHT, meaning that a further reduction in hair cell survival could not be detected. PHT did not sensitize hair cells to neomycin toxicity (**Figure 1**; one-way ANOVA, *p* = 0.17). PHT was not ototoxic on its own (“zero AAB” data points in **Figures 1D,E** and data not shown). These data suggest that either PDK1 or Akt normally protect hair cells specifically from gentamicin toxicity. Using 1 μM Akt inhibitor, which has been previously used in the zebrafish lateral line, we found that Akt-specific inhibition did not modulate aminoglycoside-induced hair cell death (Uribe et al., 2015, and **Figure 2**; two-way ANOVA, *p* = 0.13 and *p* = 0.35 for neomycin and gentamicin, respectively). By contrast, the PDK1 inhibitor BX795 significantly sensitized hair cells to 25 μM gentamicin, with a 13.7% decreased in hair cell survival in the presence of 1 μM BX795 (**Figure 3A**; one-way ANOVA, *p* < 0.001). However, this sensitization was not evident at higher gentamicin concentrations (**Figure 3B**; two-way ANOVA, *p* = 0.06). Collectively, our data suggest PDK1 as the endogenously protective component of this pathway.

**FIGURE 4:**
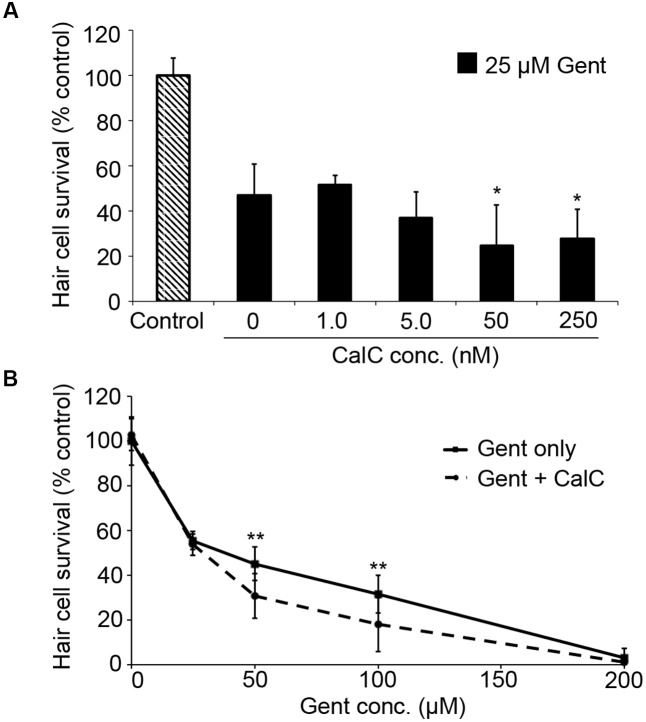
The PKC inhibitor Calphostin C (CalC) facilitates gentamicin-induced hair cell death. **(A)** CalC significantly sensitizes hair cells to damage from 25 μM gentamicin (one-way ANOVA, *F*_4,31_ = 6.16, *p* = 0.0009). Either 50 or 250 nM CalC facilitates damage (^∗^*p* < 0.05 in each case). *N* = 5–8 fish/treatment. **(B)** 250 nM CalC sensitizes hair cells to varying concentrations of gentamicin (two-way ANOVA with inhibitor presence as the factor, *F*_1,81_ = 12.48, *p* = 0.0007). Significant sensitization is seen at 50 or 100 μM gentamicin (^∗∗^*p* < 0.01). *N* = 8–10 fish/treatment.

PDK1 can activate both Akt and PKC. As our data in **Figure 3** suggest that gentamicin damage does not rely on Akt, we next assessed the role of PKC using the specific inhibitor Calphostin C (Schmidt et al., 2004). Calphostin C significantly facilitated gentamicin toxicity (**Figure 4A**; one-way ANOVA, *p* = 0.0009), with approximately a 20% reduction in hair cell survival with 50 or 250 nM Calphostin C, respectively. 250 nM Calphostin C sensitized hair cells to variable concentrations of gentamicin (two-way ANOVA, *p* = 0.0007; **Figure 4B**), with a 14.5% reduction in hair cell survival seen at 50 μM gentamicin and a 13.6% reduction observed with 100 μM gentamicin. These data suggest that PDK1 may protect hair cells by activation of PKC.

**FIGURE 5:**
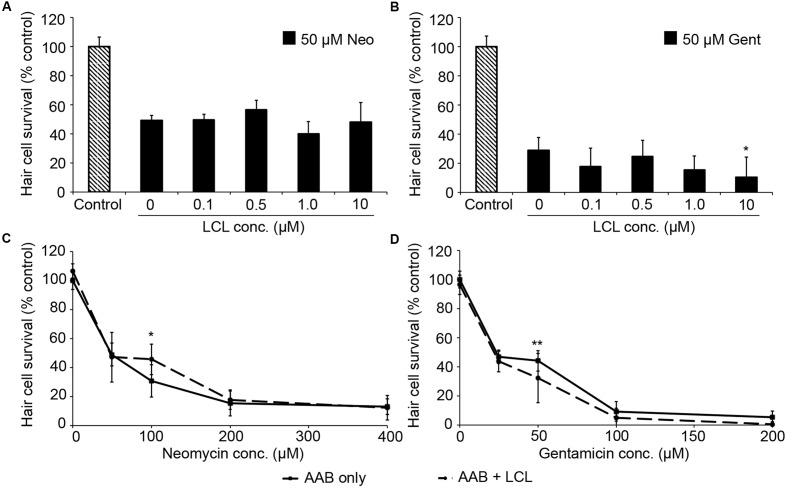
The Smac mimetic LCL-161, which inhibits cIAP1, modestly sensitizes hair cells to gentamicin damage. **(A)** Variable LCL-161 concentrations do not affect toxicity to 50 μM neomycin (one-way ANOVA, *F*_4,69_ = 1.54, *p* = 0.199). **(B)** Variable LCL has a modest but significant effect on gentamicin toxicity (one-way ANOVA, *F*_4,31_ = 3.14, *p* = 0.028), with 10 μM LCL conveying significant sensitization (^∗^*p* < 0.05). **(C)** 10 μM LCL appears to modestly protect hair cells from 100 μM neomycin (two-way ANOVA, *F*_1,74_ = 4.34, *p* = 0.041, *post hoc* analysis ^∗^*p* < 0.05). **(D)** 10 μM LCL increases susceptibility to gentamicin (two-way ANOVA with inhibitor presence as the factor, *F*_1,88_ = 12.93, *p* < 0.001). Sensitization is only apparent at 50 μM gentamicin (^∗∗^*p* < 0.01). Solid lines are aminoglycoside (AAB) only, dashed lines are aminoglycoside with LCL. *N* = 6–12 fish/treatment.

**FIGURE 6:**
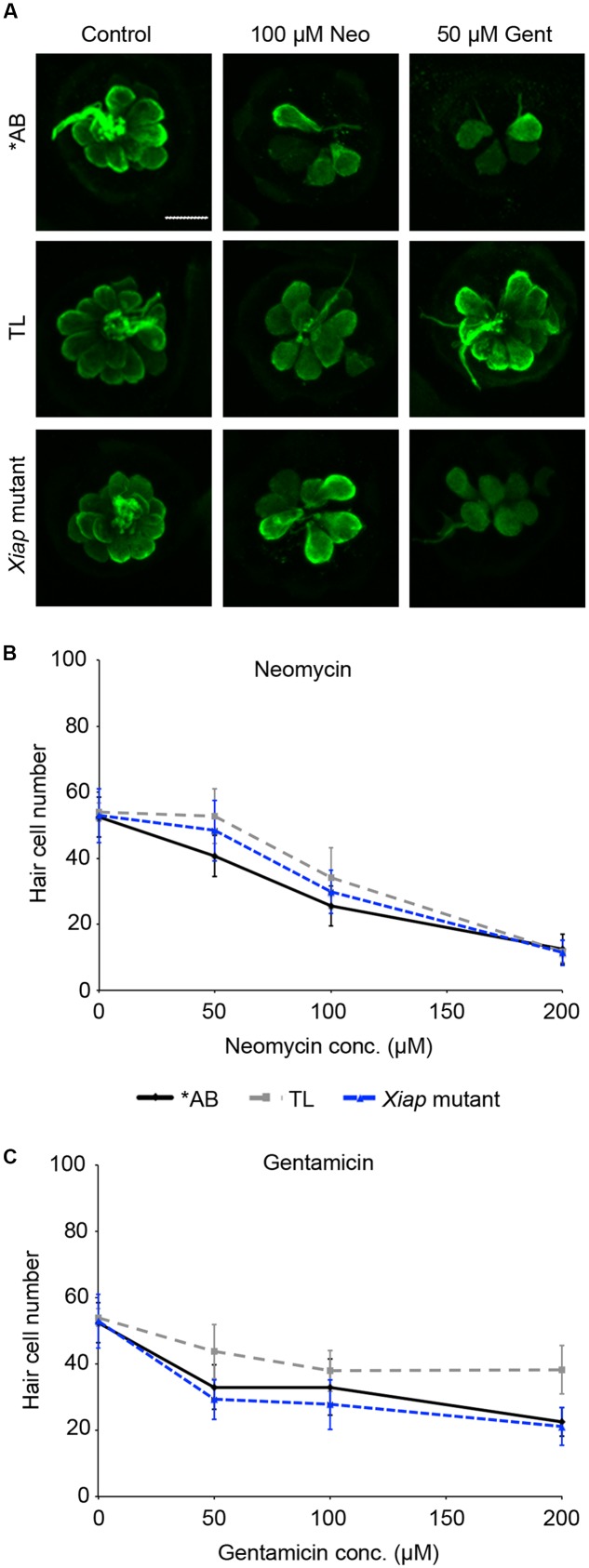
Ototoxin sensitivity differs by genotype. ^∗^AB, TL, or *Xiap* mutant fish (created on a TL background) were treated acutely with neomycin or continuously with gentamicin. Hair cells were assessed by direct counts of anti-parvalbumin-labeled cells. **(A)** Representative confocal images of anti-parvalbumin-labeled hair cells from ^∗^AB, TL, and *Xiap* mutant fish. The scale bar = 10 μm and applies to all panels. **(B)** Genotype significantly effects hair cell sensitivity to neomycin (two-way ANOVA, *F*_2,102_ = 6.363, *p* = 0.002). Significant pairwise comparisons: 50 μM neo: AB vs TL *p* < 0.001, AB vs. *Xiap p* < 0.05; 100 μM neo: AB vs. TL *p* < 0.05. **(C)** There is a significant difference of genotype on gentamicin sensitivity (two-way ANOVA with genotype as the factor, *F*_3,96_ = 24.15, *p* < 0.0001). There is also a significant interaction between genotype and gentamicin concentration (*F*_6,96_ = 3.028, *p* = 0.009). Significant pairwise comparisons: 50 μM gent: AB vs. TL *p* < 0.01, TL vs. *Xiap p* < 0.001; 100 μM gent: TL vs. *Xiap p* < 0.01; 200 μM gent: AB vs. TL *p* < 0.001, TL vs. *Xiap p* < 0.001. In panels **(B,C)**, black solid lines are ^∗^AB fish, gray dashed lines are TL fish, and blue dashed lines are *Xiap* mutant fish. *N* = 7–11 fish/treatment.

We then examined the role of IAPs in aminoglycoside toxicity. The Smac mimetic LCL-161, which targets cIAP1 and cIAP2 for degradation and leads to caspase activation, modestly sensitized hair cells to gentamicin damage (**Figure 5**; one-way ANOVA, *p* = 0.28), with 10 μM LCL reducing hair cell survival by 18.4% over gentamicin only. 10 μM LCL appeared to protect hair cells from neomycin damage, at least at 100 μM neomycin, with 14.8% more hair cells surviving when LCL was present (**Figure 5C**; two-way ANOVA, *p* = 0.041). Higher concentrations of LCL were ototoxic on their own (data not shown). More substantial results were observed in *Xiap* loss of function mutant fish. Hair cells in *Xiap* mutants were significantly more sensitive to gentamicin than were hair cells in wild type TL fish, the background strain on which the *Xiap* mutant line was created (**Figure 6**; two-way ANOVA, *p* < 0.0001). There was a modest effect of genotype on neomycin sensitivity (two-way ANOVA, *p* = 0.002). However, this effect resulted from a difference in neomycin susceptibility between ^∗^AB and TL fish, while *Xiap* mutant and TL hair cells did not differ in neomycin sensitivity (two-way ANOVA, *p* = 0.12). These data suggest that endogenous *Xiap* expression may protect hair cells from gentamicin, but not from neomycin damage. We also found that TL hair cells were resistant to gentamicin when compared to wild type ^∗^AB fish, demonstrating strain-specific differences in aminoglycoside susceptibility (**Figure 6**; see figure legend for pairwise statistical comparisons).

### Strain Differences in p53 Signaling

**FIGURE 7:**
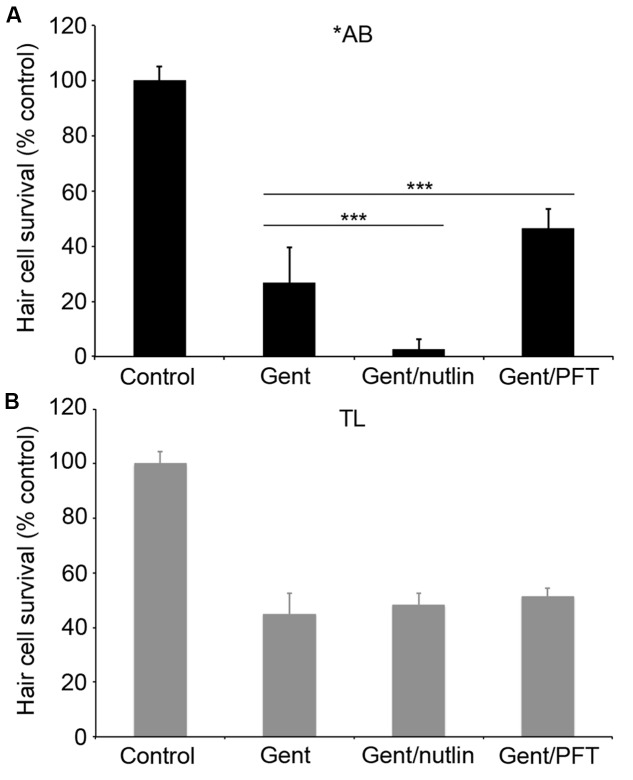
p53 modulation does not affect gentamicin-induced hair cell death in TL fish. Fish were treated with 25 μM gentamicin either alone, with **(A)** 50 μM nutlin-3a, or with **(B)** 25 μM PFTα. Data are normalized to untreated ^∗^AB fish. There is a significant effect of treatment, genotype, and the interaction between the two (*F*_3,112_ = 705.1, *F*_1,112_ = 149.7, *F*_3,112_ = 47.07 for treatment, genotype, and the interaction term, respectively, *p* < 0.001 in all cases). Asterisks denote significant differences from gentamicin-only treatment, not including controls. ^∗∗∗^*p* < 0.001. *N* = 10–20 fish/group.

We went on to explore the differences between ^∗^AB and TL strains in more detail, focusing on the more robust differences observed in gentamicin-exposed fish. Previous studies demonstrate that p53 modulates gentamicin-induced hair cell death in zebrafish (Coffin et al., 2013a,b). There was a significant effect of fish strain on the response to p53 manipulation (**Figure 7**; two-way ANOVA, *p* < 0.001). Inhibiting p53 with PFTα significantly protected ^∗^AB hair cells from gentamicin damage (**Figure 7A**; *p* < 0.0001), with PFT-treated ^∗^AB fish having 19.7% more hair cells than ^∗^ABs treated with gentamicin only. By contrast, there was no increase in hair cell survival in TL fish (*p* = 0.14). Similarly, stabilizing p53 with nutlin-3a greatly sensitized ^∗^AB hair cells to gentamicin damage, with a 23.9% reduction in hair cells in nutlin-3a-treated fish (**Figure 7**; *p* < 0.0001). Nutlin-3a did not affect gentamicin damage in TL hair cells (*p* = 0.99). These experiments suggest that p53 signaling may be altered in TL hair cells, or that gentamicin-induced hair cell death is independent of p53 in this line.

**FIGURE 8:**
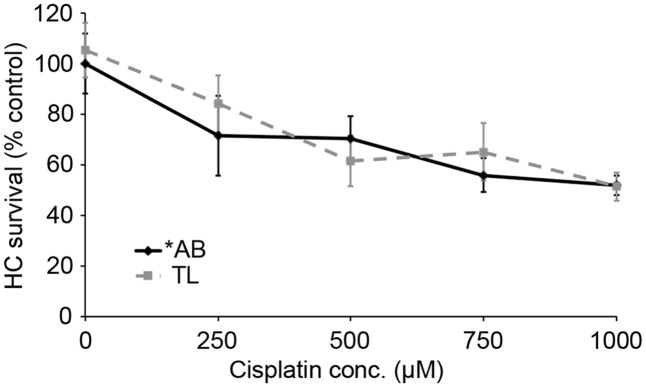
There is no strain difference in cisplatin ototoxicity. Six hours continuous exposure to cisplatin results in dose-dependent hair cell death, with no significant difference between ^∗^AB and TL fish (two-way ANOVA with genotype as a factor, *F*_1,92_ = 3.049, *p* = 0.084). *N* = 9–13 fish/treatment.

We next asked if TL hair cells are resistant to cisplatin damage. Cisplatin is an ototoxic chemotherapy drug that, in the lateral line, kills hair cells in a p53-independent manner (Ou et al., 2007; Coffin et al., 2013a). As shown in **Figure 8**, there was no difference in cisplatin-induced hair cell death between TL and ^∗^AB fish (two-way ANOVA, *p* = 0.084), suggesting that the relative resistance of TL hair cells to damage is specific for aminoglycosides and may be related to differences in p53.

**FIGURE 9:**
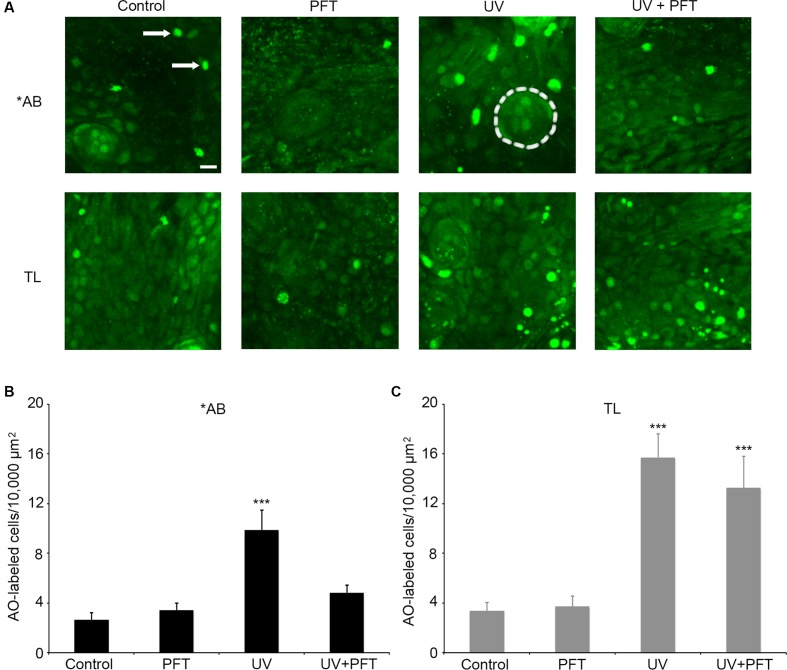
p53 differentially modulates UV-induced cell death between AB and TL fish. **(A)** Representative confocal images of the ventral head region, showing the differences in AO labeling between groups. Each box is 100 × 100 μm; the actual size of the regions used to collect the quantitative data in panels **(B,C)**. Arrows in the upper left panel point to examples of AO-labeled cells. Dashed line in the upper middle panel (AB/UV group) outlines a neuromast for reference. Scale bar in the upper left panel = 10 μm and applies to all panels. **(B)** UV exposure in ^∗^AB fish significantly kills cells, and PFTα treatment returns cell death to baseline levels. One-way ANOVA, *F*_3,25_ = 11.27, *p* < 0.001. **(C)** UV exposure also increase the number of AO-labeled cells in TL fish, and PFTα does not prevent cell death. One-way ANOVA, *F*_3,26_ = 13.96, *p* < 0.001. For both panels, asterisks “^∗∗∗^” indicate different from controls of the same strain, *p* < 0.001. *N* = 6–8 fish/group.

Finally, we asked if cell death in response to UV light exposure was altered in TL fish. UV light kills multiple cell types through p53-dependent signaling cascades (Zeng et al., 2009; Gong et al., 2015). There was a significant effect of treatment for either fish strain (**Figure 9**; one-way ANOVA, *p* < 0.001 for each strain). UV light caused a threefold increase in the number of AO-labeled cells in the ventral head region of ^∗^AB fish (*p* < 0.0001), and this increase was attenuated by treatment with PFTα, with no significant difference observed between control fish and fish exposed to UV but treated with PFTα (**Figure 9B**, *p* = 0.87). In TL fish, UV exposure also led to a significant fourfold increase in AO labeling (*p* < 0.0001), but PFTα did not significantly reduce the magnitude of cell death (**Figure 9C**; *p* = 0.99). In neither case did PFTα itself alter the number of AO-labeled cells. Collectively, these data point to strain-specific differences in p53-dependent cell death signaling.

## Discussion

### Cell Death and Survival in Aminoglycoside-Damaged Hair Cells

The goal of this study was to identity new candidate molecules involved in aminoglycoside-damaged hair cells of the zebrafish lateral line, and to experimentally manipulate them to determine their effect on hair cell death. We used DAVID and GeneMANIA to construct hypothetical genetic interaction pathways for neomycin and gentamicin damage, using known hair cell death molecules from the Bcl2 family and pharmacological modulators of aminoglycoside ototoxicity in the lateral line as the basis for selecting input genes (Coffin et al., 2013a,b). Our pathway analysis yielded several signaling proteins known to modulate aminoglycoside ototoxicity in mammalian systems, such as JNK and heat shock proteins (e.g., Wang et al., 2003; Cunningham and Brandon, 2006; Sugahara et al., 2006; Taleb et al., 2008). However, our analysis also yielded other candidate genes for future exploration. Collectively, the pathway analysis provides new molecular targets for ototoxicity research.

The pathway analysis diagrams in Supplementary Figure 1 differ for gentamicin and neomycin, because our previous research demonstrates that in the lateral line, these aminoglycosides activate only partially overlapping suites of cell death regulators (Coffin et al., 2009, 2013a,b; Owens et al., 2009). For the present study, we focused on proteins from the gentamicin analysis, as gentamicin ototoxicity is relatively understudied in the lateral line, and gentamicin is in widespread clinical use. We selected three candidates for manipulation: the PI3K signaling protein PDK1, the inhibitor of apoptosis protein Xiap, and the mitochondrial protein Smac. We found that PDK1 inhibition sensitized hair cells to gentamicin damage but not neomycin toxicity, suggesting that endogenous PDK1 may protect hair cells from gentamicin exposure. In rodent models, aminoglycoside exposure can alter expression of PI3K signaling components and PI3K inhibition sensitizes cochlear explants to gentamicin damage (Chung et al., 2006; Jiang et al., 2006b; Jadali and Kwan, 2016). Our data suggest that PDK1, which activates downstream members of the PI3K pathway, may be responsible for the PI3K-dependent modulation of aminoglycoside ototoxicity.

PDK1 can phosphorylate multiple downstream targets, including Akt and PKC (Mora et al., 2004). Akt inhibition does not alter aminoglycoside susceptibility in the lateral line (**Figure 3**; Uribe et al., 2015), suggesting that PKC or other downstream PDK1 targets are responsible for the modulatory effect we observed. In mammals, multiple studies have implicated Akt in aminoglycoside ototoxicity, although the evidence for a protective role comes primarily from neonatal cochlear cultures, which may not accurately represent the mature *in vivo* condition (Chung et al., 2006; Brand et al., 2011, 2015; Heinrich et al., 2015). Our data suggest that endogenous Akt does not attenuate aminoglycoside ototoxicity *in vivo*, and that the effect of PDK1 manipulation is dependent on PKC, consistent with *in vitro* data from Chung et al. (2006). PDK1-dependent PKC signaling mediates mechanical hypersensitivity in a neuropathic pain model, demonstrating a role for this signaling cascade in nervous system injury (Ko et al., 2016). PDK1 activation of PKC also contributes to increased cell survival in some tumor types, likely by interacting with Bcl2-family proteins (Desai et al., 2011; Zabiewicz et al., 2014). Our previous data demonstrate that over-expression of Bcl2 protects hair cells from gentamicin toxicity (Coffin et al., 2013a), and the current research suggests that PKC signaling may play a role in this protective effect.

Our data do not allow us to determine the cell type(s) specifically responsible for PI3K-dependent protection. The effect could be cell-autonomous and confined to hair cells, but previous studies demonstrate that supporting cells are critical for hair cell survival in the undamaged ear and play an important role in hair cell responses to ototoxic damage (Haddon et al., 1998; May et al., 2013; Jadali et al., 2017). PI3K signaling is dependent on an extracellular ligand binding to a membrane-bound receptor to initiate the intracellular cascade (reviewed in Gamper and Powerll, 2012; Cunningham and Ruggero, 2013). Therefore, PDK1 modulation of hair cell survival depends on an upstream signal that could originate in neighboring supporting cells, or in a potentially distant cell population. A similarly cooperative mechanism was proposed by Jadali and Kwan (2016), who suggested that IGF-1 or another excreted ligand may be necessary for PI3K-dependent hair cell protection. Future experiments are necessary to further examine PI3K signaling in ototoxicity and to determine the source of the ligand and the cell types involved.

The IAPs play roles in cell proliferation, differentiation, and survival. The Smac mimetic LCL-161, which targets cIAP1 and cIAP2 for degradation (Vince et al., 2007; West et al., 2016), modestly altered hair cell responses to aminoglycosides, with differing responses for neomycin and gentamicin. It is unclear if these modest but opposing differences are biologically relevant, and future experiments are needed to further probe the role of these IAPs in ototoxicity. However, *Xiap* loss-of-function significantly sensitized hair cells to gentamicin damage, but not to neomycin toxicity, suggesting a protective role for endogenous *Xiap* during gentamicin exposure. Xiap can modulate cell death via both caspase-dependent and caspase-independent pathways, and plays diverse roles in cellular responses to stresses such as toxin exposure (Dubrez-Daloz et al., 2008). *Xiap* over-expression protects hair cells from cisplatin damage, and modestly reduces neomycin ototoxicity in P8-P14 mice (Cooper et al., 2006; Sun et al., 2014). It is possible that *Xiap* over-expression in zebrafish would prevent neomycin-induced hair cell loss, as increasing pro-survival factors can confer protection even if endogenous levels appear insufficient to modulate hair cell damage (Uribe et al., 2015). However, our results provide further evidence for differences in cell signaling responses to neomycin vs. gentamicin in zebrafish hair cells.

Xiap can function as an ubiquitin ligase, consistent with our previous data demonstrating a role for proteasomal degradation in aminoglycoside toxicity (Galbán and Duckett, 2010; Coffin et al., 2013b). Xiap ubiquitinates multiple targets, including apoptosis-inducing factor (AIF), leading to AIF activation and caspase-independent programmed cell death (Lewis et al., 2011). In the lateral line, aminoglycoside-induced hair cell death is independent of caspase activation, implicating this caspase-independent role of Xiap in gentamicin damage (Coffin et al., 2013b). AIF translocates from the mitochondria to the nucleus in damaged cells, and AIF translocation is reported in hair cells in response to amikacin administration *in vivo* or to gentamicin *in vitro*, suggesting that AIF may mediate hair cell damage (Lecain et al., 2007; Zhang et al., 2012). However, an *in vivo* study in mice did not find AIF translocation after kanamycin ototoxicity, but did note a decrease in cochlear AIF expression (Jiang et al., 2006a). It is unclear if these differences are due to species differences or to the aminoglycoside used. Future research is necessary to determine if an Xiap–AIF interaction plays a role in aminoglycoside ototoxicity in the lateral line.

GeneMANIA predicts interactions between genes, yielding potential candidates for experimental follow-up. Our bioinformatics analysis of the different cell signaling genes associated with aminoglycoside damage helped identify potential molecular targets for modulation of hair cell responses to neomycin or gentamicin. Both *PDK1* and *Xiap* appeared on the gentamicin signaling diagram but not the neomycin diagram, and modulation of PDK1 or Xiap influenced hair cell death in response to gentamicin but not neomycin. These data further highlight the toxin-specific nature of hair cell damage, and the utility of the bioinformatics approach to identify potential candidate molecules for experimental validation.

### Strain-Specific Responses to Aminoglycoside Damage

During our cell signaling investigation, we found strain-specific differences in aminoglycoside sensitivity, with TL fish being modestly resistant to neomycin-induced hair cell death, and substantially resistant to gentamicin damage. Our previous work demonstrated that p53 inhibition conferred modest protection from neomycin and substantial protection from gentamicin in ^∗^AB fish (Coffin et al., 2013a). We therefore investigated p53 modulation in ^∗^AB vs. TL fish here and found that p53 modulation did not alter hair cell responses to gentamicin in TL fish, in contrast to the marked effect on hair cell death in ^∗^AB animals. Our p53 manipulation experiments suggest that ∼40% of gentamicin-induced hair cell death in the lateral line is independent of p53, since in ^∗^AB fish p53 inhibition partially rescues gentamicin damage, leading to hair cell loss on par with gentamicin-treated TL hair cells that did not receive p53 inhibitor. Cell death due to UV exposure is also p53-dependent, and UV exposure induced cell death in both ^∗^AB and TL larvae. p53 inhibition attenuated damage in ^∗^AB fish but not in TLs, further evidence for strain-specific differences in p53 signaling, leading to strain-specific responses to damaging agents.

Strain-specific differences in aminoglycoside sensitivity were previously noted in rodents (Sullivan et al., 1987; Wu et al., 2001). For example, Fisher-344 rats are more susceptible to gentamicin ototoxicity and nephrotoxicity than are Sprague-Dawley rats, and BALB/c mice are more susceptible to kanamycin damage than C57BL/6 mice (Sullivan et al., 1987; Wu et al., 2001). Human genetic differences in ototoxic sensitivity are well-known, particularly mitochondrial mutations that convey increased susceptibility to aminoglycoside-induced hearing loss (Fischel-Ghodsian, 1998). To our knowledge, this is the first report of a strain difference in aminoglycoside susceptibility in zebrafish. Monroe et al. (2016) demonstrated that different strains of adult zebrafish differ in hearing sensitivity, with TL fish having greater sensitivity than age-matched ^∗^ABs. However, the cause of these differences is unknown.

Zebrafish exhibit strain-specific responses to several behavioral tasks, such as inhibitory avoidance learning or startle response habituation (Gorissen et al., 2015; van den Bos et al., 2017). TL fish have reduced resting cortisol levels and decreased expression of stress-related genes when compared to AB fish, and our unpublished results suggest that cortisol may increase aminoglycoside susceptibility in lateral line hair cells (Gorissen et al., 2015; van den Bos et al., 2017; Hayward and Young, unpublished data). Therefore, in addition to differences in p53 signaling, increased cortisol levels in ^∗^AB animals may further sensitize their hair cells to aminoglycoside damage. Future experiments are needed to dissect the relative contributions of stress-related factors and strain-specific aminoglycoside sensitivity.

## Conclusion

Our pathway analysis suggests several novel candidates for modulation of aminoglycoside ototoxicity, and our pharmacological and genetic manipulations demonstrate likely roles for the PI3K proteins PDK1 and PKC, and the IAPs Xiap, as endogenous regulators of gentamicin toxicity. We further demonstrate that strain-specific differences in p53 signaling likely underlie differential susceptibility to gentamicin damage. These studies increase understanding of cell signaling in ototoxic damage and suggest molecular targets for possible therapeutic intervention.

## Author Contributions

HW, LH, and AC all participated in the research design, conducted experiments, performed data analysis, and wrote or contributed to the writing of this manuscript.

## Conflict of Interest Statement

The authors declare that the research was conducted in the absence of any commercial or financial relationships that could be construed as a potential conflict of interest.
