# Supplementary material for: PI3K and Inhibitor of Apoptosis Proteins Modulate Gentamicin- Induced Hair Cell Death in the Zebrafish Lateral Line
Source: Front Cell Neurosci. 2017 Oct 18;11:326. doi: 10.3389/fncel.2017.00326 (PMC5651234; doi:10.3389/fncel.2017.00326)
Supplement: Supplementary file 2 [file Data_Sheet_1.PDF]

GeneMANIA report

Created on : 22 July 2017 18:54:46
Last database update : 13 March 2017 00:00:00
Application version : 3.5.0

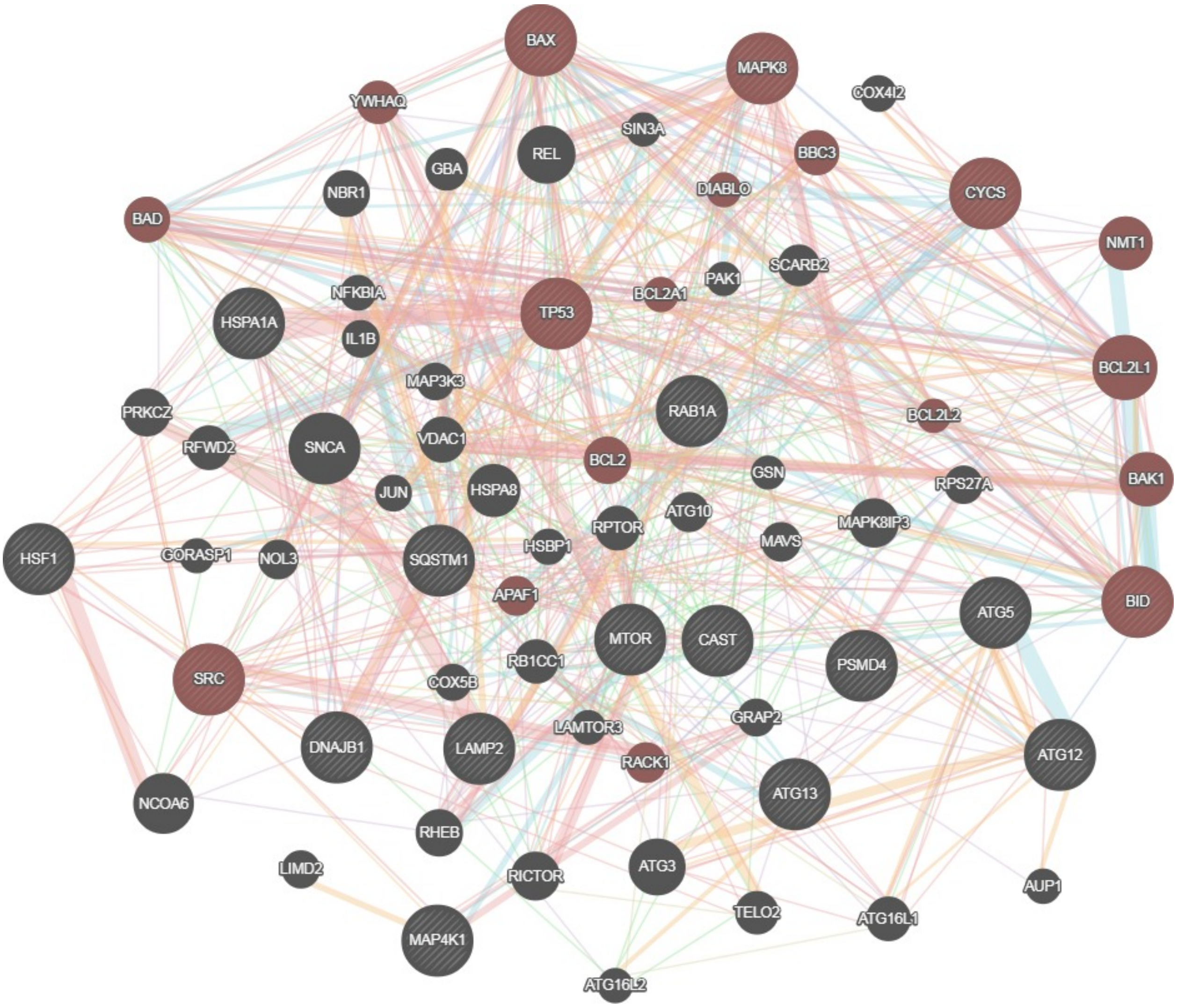

Networks

- Physical Interactions
- Pathway
- Co-expression
- Predicted
- Co-localization
- Genetic Interactions
- Shared protein domains

Functions

- intrinsic apoptotic signaling pathway

Search parameters

Organism Homo sapiens (human)

Genes 3920 , 637 , 581 , 11184 , 5599 , 3337 , 5861 , 7157 , 9140 , 831 , 5710 , 8878 , 9776 , 3297 , 6714 , 2475 , 3303 , ATG5 , CYCS

Network weighting Automatically selected weighting method

Networks

A

Abu-Odeh-Aqeilan-2014 , Agrawal-Sedivy-2010 , Aichem-Groettrup-2012 , Albers-Koegl-2005 , Alexandru-Deshaies-2008 , Alizadeh-Staudt-2000 , Andresen-Flores-Morales-2014 , Arbuckle-Grant-2010 , Arroyo-Aloy-2014 , Arroyo-Aloy-2015

B

Bahr-Bowler-2013 , Bailey-Hieter-2015 , Bandyopadhyay-Ideker-2010 , Bantscheff-Drewes-2011 , Barr-Knapp-2009 , Barrios-Rodiles-Wrana-2005 , Behrends-Harper-2010 , Behzadnia-Lührmann-2007 , Bennett-Harper-2010 , Benzinger-Hermeking-2005 , Berggård-James-2006 , Bett-Hay-2013 , Bhatnagar-Attie-2014 , Bild-Nevins-2006 B , BIOGRID-SMALL-SCALE-STUDIES , BIOGRID-SMALL-SCALE-STUDIES , Blandin-Richard-2013 , Blomen-Brummelkamp-2015 , Blomen-Brummelkamp-2015 , Bogachek-Weigel-2014 , Boldrick-Relman-2002 , Bonacci-Soubeyran-2014 , Bouwmeester-Superti-Furga-2004 , Brajenovic-Drewes-2004 , Brehme-Superti-Furga-2009 , Bruderer-Hay-2011 , Burington-Shaughnessy-2008 , Butland-Hayden-2014 , Byron-Humphries-2012

C

Cai-Conaway-2007 , Camargo-Brandon-2007 , Campos-Reinberg-2015 , Cao-Chinnaiyan-2014 , Carmon-Liu-2014 , CELL_MAP , Chen-Brown-2002 , Chen-Ge-2013 , Chen-Huang-2014 , Chen-Zhang-2013 , Christianson-Kopito-2011 , Cloutier-Coulombe-2013 , Colland-Gauthier-2004 , Corominas-Iakoucheva-2014 , Couzens-Gingras-2013 , Cox-Rizzino-2013 , Coyaud-Raught-2015

D

Danielsen-Nielsen-2011 , Dart-Wells-2015 , de Hoog-Mann-2004 , Diner-Cristea-2015 , Dobbin-Giordano-2005 , Drissi-Boisvert-2015 , Dyer-Sobral-2010

E

Emanuele-Elledge-2011 , Emdal-Olsen-2015 , Ewing-Figeys-2007

F

Fenner-Prehn-2010 , Floyd-Pagliarini-2016 , Foerster-Ritter-2013 , Fogeron-Lange-2013 , Foster-Marshall-2013 , Freibaum-Taylor-2010

G

Gabriel-Baumgrass-2016 , Galligan-Howley-2015 , Gao-Reinberg-2012 , Gautier-

G

Hall-2009 , Giannone-Liu-2010 , Glatter-Gstaiger-2009 , Gloeckner-Ueffing-2007 , Goehler-Wanker-2004 , Golebiowski-Hay-2009 , Goudreault-Gingras-2009 , Grant-2010 , Greco-Cristea-2011 , Grossmann-Stelzl-2015 , Guarani-Harper-2014 , Gupta-Pelletier-2015

H

Hanson-Clayton-2014 , Hauri-Gstaiger-2013 , Havrylov-Redowicz-2009 , Havugimana-Emili-2012 , Hayes-Urbé-2012 , Hegele-Stelzl-2012 A , Hegele-Stelzl-2012 B , Hein-Mann-2015 , Hill-Livingston-2014 , HUMANCYC , Humphries-Humphries-2009 , Hutchins-Peters-2010 , Huttlin-Gygi-2015

I

I2D-BIND-Fly2Human , I2D-BIND-Mouse2Human , I2D-BIND-Rat2Human , I2D-BIND-Worm2Human , I2D-BIND-Yeast2Human , I2D-BioGRID-Fly2Human , I2D-BioGRID-Mouse2Human , I2D-BioGRID-Rat2Human , I2D-BioGRID-Worm2Human , I2D-BioGRID-Yeast2Human , I2D-Chen-Pawson-2009-PiwiScreen-Mouse2Human , I2D-Formstecher-Daviet-2005-Embryo-Fly2Human , I2D-Giot-Rothbert-2003-Low-Fly2Human , I2D-INNATEDB-Mouse2Human , I2D-IntAct-Fly2Human , I2D-IntAct-Mouse2Human , I2D-IntAct-Rat2Human , I2D-IntAct-Worm2Human , I2D-IntAct-Yeast2Human , I2D-Krogan-Greenblatt-2006-Core-Yeast2Human , I2D-Krogan-Greenblatt-2006-NonCore-Yeast2Human , I2D-Li-Vidal-2004-CORE-1-Worm2Human , I2D-Li-Vidal-2004-non-core-Worm2Human , I2D-Manual-Mouse2Human , I2D-Manual-Rat2Human , I2D-MGI-Mouse2Human , I2D-MINT-Fly2Human , I2D-MINT-Mouse2Human , I2D-MINT-Rat2Human , I2D-MINT-Worm2Human , I2D-MINT-Yeast2Human , I2D-Ptacek-Snyder-2005-Yeast2Human , I2D-Tarassov-PCA-Yeast2Human , I2D-Tewari-Vidal-2004-TGFb-Worm2Human , I2D-vonMering-Bork-2002-High-Yeast2Human , I2D-vonMering-Bork-2002-Low-Yeast2Human , I2D-vonMering-Bork-2002-Medium-Yeast2Human , I2D-Wang-Orkin-2006-EScmplx-Mouse2Human , I2D-Wang-Orkin-2006-EScmplxlow-Mouse2Human , I2D-Yu-Vidal-2008-GoldStd-Yeast2Human , IMID , Ingham-Pawson-2005 , Innocenti-Brown-2011 , INTERPRO , IREF-BIND , IREF-BIOGRID , IREF-DIP , IREF-HPRD , IREF-INTACT , IREF-MATRIXDB , IREF-MPPI , IREF-PUBMED , IREF-SMALL-SCALE-STUDIES , IREF-SMALL-SCALE-STUDIES

J

Jeronimo-Coulombe-2007 , Jin-Pawson-2004 , Johnson-Kerner-Wichterle-2015 , Johnson-Shoemaker-2003 , Jones-MacBeath-2006 , Joshi-Cristea-2013 , Jäger-Krogan-2011

K

Kahle-Zoghbi-2011 , Kaltenbach-Hughes-2007 , Katsogiannou-Rocchi-2014 , Kim-Gygi-2011 , Kim-Major-2015 , Kneissl-Grummt-2003 , Koch-Hermeking-2007 , Kotlyar-Jurisica-2015 , Kristensen-Foster-2012 , Kärblane-Sarmiento-2015 , Kırlı-

K

Görlich-2015

L

Lambert-Gingras-2015 , Lamoliatte-Thibault-2014 , Lau-Ronai-2012 , Lee-Songyang-2011 , Lehner-Sanderson-2004 A , Lehner-Sanderson-2004 B , Leng-Wang-2014 , Leung-Jones-2014 , Li-Chen-2015 , Li-Dorf-2011 A , Li-Dorf-2011 B , Li-Dorf-2014 , Li-Haura-2013 , Lim-Zoghbi-2006 , Lin-Smith-2010 , Lipp-Guthrie-2015 , Liu-Wang-2012 , Llères-Lamond-2010 , Loch-Strickler-2012 , Low-Heck-2014 , Lu-Zhang-2013 , Luo-Elledge-2009

M

Mak-Moffat-2010 , Mallon-McKay-2013 , Malovannaya-Qin-2010 , Markson-Sanderson-2009 , Maréchal-Zou-2014 , Matsumoto-Nakayama-2005 , McCracken-Blencowe-2005 , McFarland-Nussbaum-2008 , Meek-Piwnica-Worms-2004 , Milev-Mouland-2012 , Miyamoto-Sato-Yanagawa-2010 , Murakawa-Landthaler-2015

N

Nakayama-Ohara-2002 , Nakayasu-Adkins-2013 , Napolitano-Meroni-2011 , Narayan-Bennett-2012 , Nathan-Goldberg-2013 , NCI_NATURE , Neganova-Lako-2011 , Newman-Keating-2003 , Nicholson-Hupp-2014 , Noble-Diehl-2008

O

Oliviero-Cagney-2015 , Olma-Pintard-2009 , Oláh-Ovádi-2011 , Oshikawa-Nakayama-2012 , Ouyang-Gill-2009

P

Panigrahi-Pati-2012 , Papp-Lamia-2015 , Perez-Hernandez-Yáñez-Mó-2013 , Perou-Botstein-1999 , Perou-Botstein-2000 , Persaud-Rotin-2009 , Petschnigg-Stagljar-2014 , PFAM , Phillips-Corn-2013 , Pichlmair-Superti-Furga-2011 , Pichlmair-Superti-Furga-2012 , Pilot-Storck-Goillot-2010 , Povlsen-Choudhary-2012

R

Ramachandran-LaBaer-2004 , Raman-Harper-2015 , Ramaswamy-Golub-2001 , Ravasi-Hayashizaki-2010 , REACTOME , Reinke-Keating-2013 , Reyniers-Taymans-2014 , Richter-Chrzanowska-Lightowlers-2010 , Rieger-Chu-2004 , Rolland-Vidal-2014 , Rosenwald-Staudt-2001 , Roth-Zlotnik-2006 , Roux-Burke-2012 , Rowbotham-Mermoud-2011 , Roy-Pardo-2014 , Roy-Parent-2013 , Rual-Vidal-2005 A , Rual-Vidal-2005 B

S

Sang-Jackson-2011 , Sato-Conaway-2004 , Schadt-Shoemaker-2004 , Scholz-Taylor-2016 , Singh-Moore-2012 , Smirnov-Cheung-2009 , So-Colwill-2015 , Soler-López-Aloy-2011 , Sowa-Harper-2009 , Stehling-Lill-2012 , Stehling-Lill-2013 , Stelzl-Wanker-2005 , Stes-Gevaert-2014 , Stuart-Kim-2003 , Suter-Wanker-2013

T

T

Taipale-Lindquist-2012 , Taipale-Lindquist-2014 , Takahashi-Conaway-2011 , Tarallo-Weisz-2011 , Tatham-Hay-2011 , Teixeira-Gomes-2010 , Thalappilly-Dusetti-2008 , Thompson-Luchansky-2014 , Tong-Moran-2014 , Toyoshima-Grandori-2012 , Tsai-Cristea-2012

U

Udeshi-Carr-2012

V

van Wijk-Timmers-2009 , Vandamme-Angrand-2011 , Varjosalo-Gstaiger-2013 , Varjosalo-Superti-Furga-2013 , Venkatesan-Vidal-2009 , Vermeulen-Mann-2010 , Vinayagam-Wanker-2011 , Virok-Fülöp-2011 , Vizeacoumar-Moffat-2013

W

Wagner-Choudhary-2011 , Wallach-Kramer-2013 , Wan-Emili-2015 , Wang-Balch-2006 , Wang-Cheung-2015 , Wang-He-2008 , Wang-Maris-2006 , Wang-Xu-2015 , Wang-Yang-2011 , Weimann-Stelzl-2013 A , Weimann-Stelzl-2013 B , Weinmann-Meister-2009 , Wen-Wu-2014 , Whisenant-Salomon-2015 , Wilker-Yaffe-2007 , Willingham-Muchowski-2003 , Witt-Labeit-2008 , Wong-O'Bryan-2012 , Woods-Monteiro-2012 , Woodsmith-Sanderson-2012 , Wu-Garvey-2007 , Wu-Li-2007 , Wu-Ma-2012 , Wu-Stein-2010 , Wu-Stein-2010

X

Xiao-Lefkowitz-2007 , Xie-Cong-2013 , Xie-Green-2012 , Xu-Ye-2012

Y

Yang-Chen-2010 , Yatim-Benkirane-2012 , Yu-Chow-2013 , Yu-Vidal-2011

Z

Zanon-Pichler-2013 , Zhang-Shang-2006 , Zhang-Zou-2011 , Zhao-Krug-2005 , Zhao-Yang-2011 , Zhou-Conrads-2004 , Zhou-Hanemann-2016

Genes

Gene	Description	Rank
ATG5	autophagy related 5 [Source:HGNC Symbol;Acc:HGNC:589]	N/A
ATG13	autophagy related 13 [Source:HGNC Symbol;Acc:HGNC:29091]	N/A
CAST	calpastatin [Source:HGNC Symbol;Acc:HGNC:1515]	N/A
HSPA1A	heat shock protein family A (Hsp70) member 1A [Source:HGNC Symbol; Acc:HGNC:5232]	N/A
ATG12	autophagy related 12 [Source:HGNC Symbol;Acc:HGNC:588]	N/A
DNAJB1	DnaJ heat shock protein family (Hsp40) member B1 [Source:HGNC Symbol;Acc:HGNC:5270]	N/A
HSF1	heat shock transcription factor 1 [Source:HGNC Symbol;Acc:HGNC:5224]	N/A
LAMP2	lysosomal associated membrane protein 2 [Source:HGNC Symbol;Acc: HGNC:6501]	N/A
RAB1A	RAB1A, member RAS oncogene family [Source:HGNC Symbol;Acc: HGNC:9758]	N/A
MAP4K1	mitogen-activated protein kinase kinase kinase kinase 1 [Source:HGNC Symbol;Acc:HGNC:6863]	N/A
BAX	BCL2 associated X protein [Source:HGNC Symbol;Acc:HGNC:959]	N/A
SQSTM1	sequestosome 1 [Source:HGNC Symbol;Acc:HGNC:11280]	N/A
BID	BH3 interacting domain death agonist [Source:HGNC Symbol;Acc: HGNC:1050]	N/A
CYCS	cytochrome c, somatic [Source:HGNC Symbol;Acc:HGNC:19986]	N/A
PSMD4	proteasome 26S subunit, non-ATPase 4 [Source:HGNC Symbol;Acc: HGNC:9561]	N/A
MTOR	mechanistic target of rapamycin [Source:HGNC Symbol;Acc:HGNC:3942]	N/A
MAPK8	mitogen-activated protein kinase 8 [Source:HGNC Symbol;Acc:HGNC: 6881]	N/A
SRC	SRC proto-oncogene, non-receptor tyrosine kinase [Source:HGNC Symbol;Acc:HGNC:11283]	N/A
TP53	tumor protein p53 [Source:HGNC Symbol;Acc:HGNC:11998]	N/A
SNCA	synuclein alpha [Source:HGNC Symbol;Acc:HGNC:11138]	1
BCL2L1	BCL2 like 1 [Source:HGNC Symbol;Acc:HGNC:992]	2
NCOA6	nuclear receptor coactivator 6 [Source:HGNC Symbol;Acc:HGNC:15936]	3
REL	REL proto-oncogene, NF-kB subunit [Source:HGNC Symbol;Acc:HGNC: 9954]	4

Gene	Description	Rank
ATG3	autophagy related 3 [Source:HGNC Symbol;Acc:HGNC:20962]	5
BAK1	BCL2 antagonist/killer 1 [Source:HGNC Symbol;Acc:HGNC:949]	6
NMT1	N-myristoyltransferase 1 [Source:HGNC Symbol;Acc:HGNC:7857]	7
HSPA8	heat shock protein family A (Hsp70) member 8 [Source:HGNC Symbol; Acc:HGNC:5241]	8
MAPK8IP3	mitogen-activated protein kinase 8 interacting protein 3 [Source:HGNC Symbol;Acc:HGNC:6884]	9
RICTOR	RPTOR independent companion of MTOR complex 2 [Source:HGNC Symbol;Acc:HGNC:28611]	10
PRKCZ	protein kinase C zeta [Source:HGNC Symbol;Acc:HGNC:9412]	11
RHEB	Ras homolog enriched in brain [Source:HGNC Symbol;Acc:HGNC:10011]	12
BCL2	B-cell CLL/lymphoma 2 [Source:HGNC Symbol;Acc:HGNC:990]	13
NBR1	NBR1, autophagy cargo receptor [Source:HGNC Symbol;Acc:HGNC: 6746]	14
VDAC1	voltage dependent anion channel 1 [Source:HGNC Symbol;Acc:HGNC: 12669]	15
BAD	BCL2 associated agonist of cell death [Source:HGNC Symbol;Acc:HGNC: 936]	16
BBC3	BCL2 binding component 3 [Source:HGNC Symbol;Acc:HGNC:17868]	17
RPTOR	regulatory associated protein of MTOR complex 1 [Source:HGNC Symbol;Acc:HGNC:30287]	18
RFWD2	ring finger and WD repeat domain 2 [Source:HGNC Symbol;Acc:HGNC: 17440]	19
ATG16L1	autophagy related 16 like 1 [Source:HGNC Symbol;Acc:HGNC:21498]	20
RB1CC1	RB1 inducible coiled-coil 1 [Source:HGNC Symbol;Acc:HGNC:15574]	21
TELO2	telomere maintenance 2 [Source:HGNC Symbol;Acc:HGNC:29099]	22
YWHAQ	tyrosine 3-monooxygenase/tryptophan 5-monooxygenase activation protein theta [Source:HGNC Symbol;Acc:HGNC:12854]	23
GBA	glucosylceramidase beta [Source:HGNC Symbol;Acc:HGNC:4177]	24
SCARB2	scavenger receptor class B member 2 [Source:HGNC Symbol;Acc:HGNC: 1665]	25
RACK1	receptor for activated C kinase 1 [Source:HGNC Symbol;Acc:HGNC: 4399]	26
ATG10	autophagy related 10 [Source:HGNC Symbol;Acc:HGNC:20315]	27
NOL3	nucleolar protein 3 [Source:HGNC Symbol;Acc:HGNC:7869]	28
MAVS	mitochondrial antiviral signaling protein [Source:HGNC Symbol;Acc:	29

Gene	Description	Rank
	HGNC:29233]	
APAF1	apoptotic peptidase activating factor 1 [Source:HGNC Symbol;Acc: HGNC:576]	30
RPS27A	ribosomal protein S27a [Source:HGNC Symbol;Acc:HGNC:10417]	31
LIMD2	LIM domain containing 2 [Source:HGNC Symbol;Acc:HGNC:28142]	32
GRAP2	GRB2-related adaptor protein 2 [Source:HGNC Symbol;Acc:HGNC:4563]	33
IL1B	interleukin 1 beta [Source:HGNC Symbol;Acc:HGNC:5992]	34
MAP3K3	mitogen-activated protein kinase kinase kinase 3 [Source:HGNC Symbol; Acc:HGNC:6855]	35
COX5B	cytochrome c oxidase subunit 5B [Source:HGNC Symbol;Acc:HGNC: 2269]	36
JUN	Jun proto-oncogene, AP-1 transcription factor subunit [Source:HGNC Symbol;Acc:HGNC:6204]	37
COX4I2	cytochrome c oxidase subunit 4I2 [Source:HGNC Symbol;Acc:HGNC: 16232]	38
HSBP1	heat shock factor binding protein 1 [Source:HGNC Symbol;Acc:HGNC: 5203]	39
NFKBIA	NFKB inhibitor alpha [Source:HGNC Symbol;Acc:HGNC:7797]	40
AUP1	ancient ubiquitous protein 1 [Source:HGNC Symbol;Acc:HGNC:891]	41
ATG16L2	autophagy related 16 like 2 [Source:HGNC Symbol;Acc:HGNC:25464]	42
BCL2A1	BCL2 related protein A1 [Source:HGNC Symbol;Acc:HGNC:991]	43
GORASP1	golgi reassembly stacking protein 1 [Source:HGNC Symbol;Acc:HGNC: 16769]	44
DIABLO	diablo IAP-binding mitochondrial protein [Source:HGNC Symbol;Acc: HGNC:21528]	45
LAMTOR3	late endosomal/lysosomal adaptor, MAPK and MTOR activator 3 [Source:HGNC Symbol;Acc:HGNC:15606]	46
SIN3A	SIN3 transcription regulator family member A [Source:HGNC Symbol; Acc:HGNC:19353]	47
PAK1	p21 (RAC1) activated kinase 1 [Source:HGNC Symbol;Acc:HGNC:8590]	48
GSN	gelsolin [Source:HGNC Symbol;Acc:HGNC:4620]	49
BCL2L2	BCL2 like 2 [Source:HGNC Symbol;Acc:HGNC:995]	50

Networks

**Physical Interactions**	**31.14%**
IREF-BIND<br>Physical Interactions with 3,659 interactions from iRefIndex	11.91%
IREF-MPPI<br>Physical Interactions with 382 interactions from iRefIndex	7.94%
Wu-Li-2007<br>Systematic identification of SH3 domain-mediated human protein-protein interactions by peptide array target screening. Wu et al (2007). *Proteomics*<br>Physical Interactions with 927 interactions from iRefIndex	7.45%
IREF-HPRD<br>Physical Interactions with 34,206 interactions from iRefIndex	1.89%
BIOGRID-SMALL-SCALE-STUDIES<br>Physical Interactions with 58,871 interactions from BioGRID	1.41%
IREF-BIOGRID<br>Physical Interactions with 155,470 interactions from iRefIndex	0.53%

**Pathway**	**28.23%**
REACTOME<br>Pathway with 24,913 interactions from Pathway Commons	14.19%
NCI_NATURE<br>Pathway with 10,122 interactions from Pathway Commons	7.97%
IMID<br>Pathway with 1,073 interactions from Pathway Commons	6.07%

**Co-expression**	**16.97%**
Noble-Diehl-2008<br>Regional variation in gene expression in the healthy colon is dysregulated in ulcerative colitis. Noble et al (2008). *Gut*<br>Co-expression with 661,539 interactions from GEO	3.83%
Rieger-Chu-2004<br>Toxicity from radiation therapy associated with abnormal transcriptional responses to DNA damage. Rieger et al (2004). *Proc Natl Acad Sci U S A*<br>Co-expression with 259,974 interactions from GEO	3.74%
Bild-Nevins-2006 B<br>Oncogenic pathway signatures in human cancers as a guide to targeted therapies. Bild et al (2006). *Nature*<br>Co-expression with 280,683 interactions from GEO	3.03%
Mallon-McKay-2013<br>StemCellDB: the human pluripotent stem cell database at the National Institutes of Health. Mallon et al (2013). *Stem Cell Res*<br>Co-expression with 585,265 interactions from GEO	1.89%

Co-expression 16.97%

Ramaswamy-Golub-2001 1.85%

Multiclass cancer diagnosis using tumor gene expression signatures. Ramaswamy et al (2001). *Proc Natl Acad Sci U S A*

Co-expression with 275,113 interactions from supplementary material

Rosenwald-Staudt-2001 1.66%

Relation of gene expression phenotype to immunoglobulin mutation genotype in B cell chronic lymphocytic leukemia. Rosenwald et al (2001). *J Exp Med*

Co-expression with 114,694 interactions from supplementary material

Innocenti-Brown-2011 0.85%

Identification, replication, and functional fine-mapping of expression quantitative trait loci in primary human liver tissue. Innocenti et al (2011). *PLoS Genet*

Co-expression with 603,765 interactions from GEO

Wang-Cheung-2015 0.11%

Genetic variation in insulin-induced kinase signaling. Wang et al (2015). *Mol Syst Biol*

Co-expression with 411,047 interactions from GEO

Predicted 13.00%

I2D-IntAct-Mouse2Human 7.58%

The IntAct molecular interaction database in 2010. Aranda et al (2010). *Nucleic Acids Res*

Predicted with 3,427 interactions from I2D

Wu-Stein-2010 4.32%

A human functional protein interaction network and its application to cancer data analysis. Wu et al (2010). *Genome Biol*

Predicted with 87,829 interactions from supplementary material

I2D-BioGRID-Yeast2Human 1.09%

BioGRID: a general repository for interaction datasets. Stark et al (2006). *Nucleic Acids Res*

Predicted with 13,434 interactions from I2D

Co-localization 8.80%

Chen-Huang-2014 8.36%

Using an in situ proximity ligation assay to systematically profile endogenous protein-protein interactions in a pathway network. Chen et al (2014). *J Proteome Res*

Co-localization with 559 interactions from BioGRID

Johnson-Shoemaker-2003 0.44%

Genome-wide survey of human alternative pre-mRNA splicing with exon junction microarrays. Johnson et al (2003). *Science*

Co-localization with 426,332 interactions from GEO

Genetic Interactions 1.54%

Lin-Smith-2010 1.54%

A genome-wide map of human genetic interactions inferred from radiation hybrid genotypes. Lin et al (2010). *Genome Res*

Genetic Interactions with 4,820,370 interactions from supplementary material

Shared protein domains 0.32%

INTERPRO 0.32%

Shared protein domains with 608,863 interactions from InterPro
