# Supplementary material for: PI3K and Inhibitor of Apoptosis Proteins Modulate Gentamicin- Induced Hair Cell Death in the Zebrafish Lateral Line
Source: Front Cell Neurosci. 2017 Oct 18;11:326. doi: 10.3389/fncel.2017.00326 (PMC5651234; doi:10.3389/fncel.2017.00326)
Supplement: Supplementary file 3 [file Data_Sheet_2.PDF]

GeneMANIA report

Created on : 23 July 2017 18:52:24
Last database update : 13 March 2017 00:00:00
Application version : 3.5.0

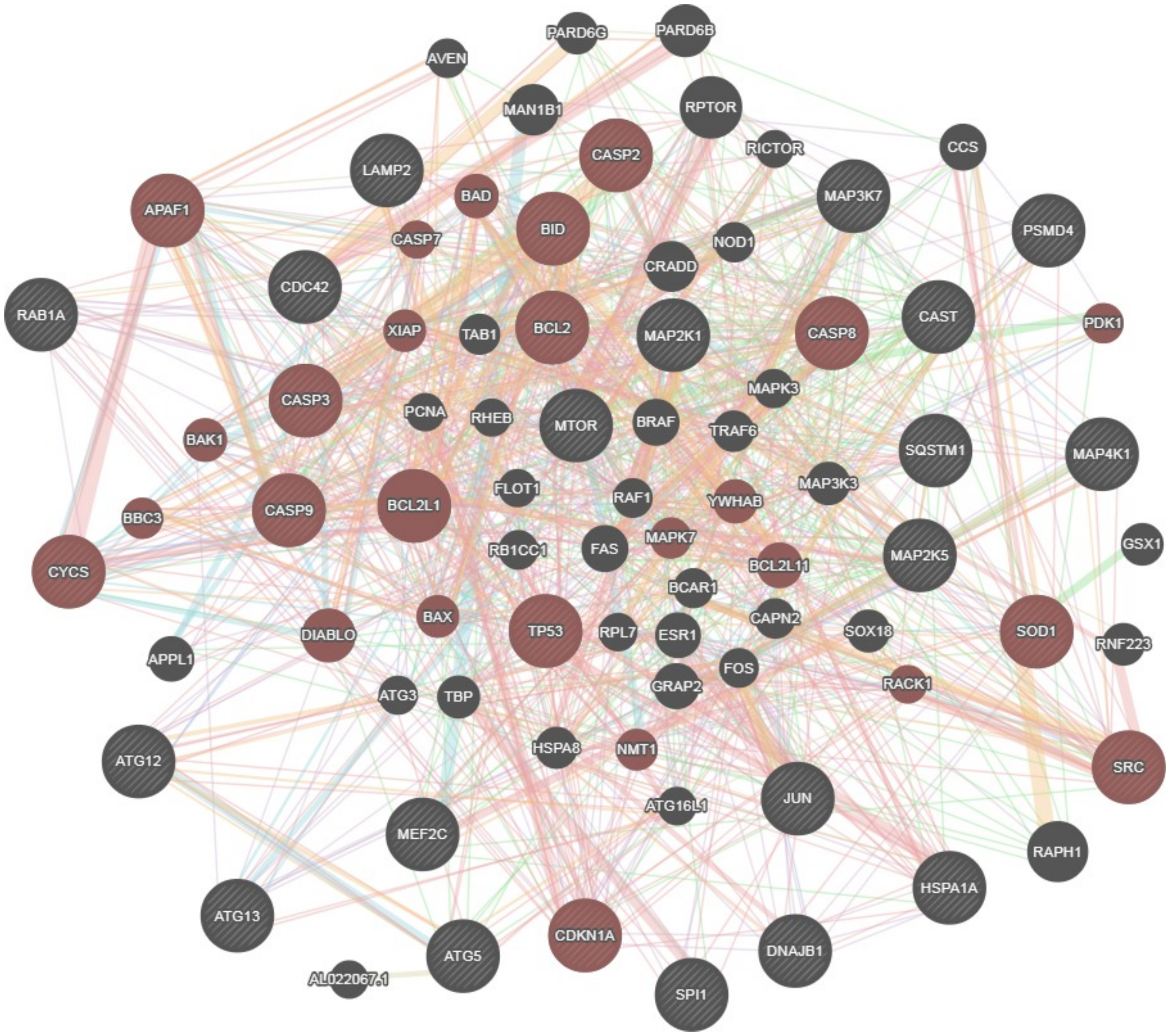

Networks

- Physical Interactions
- Predicted
- Co-expression
- Pathway
- Genetic Interactions
- Co-localization
- Shared protein domains

Functions

- intrinsic apoptotic signaling pathway

Search parameters

Organism Homo sapiens (human)

Genes 3920 , 637 , 841 , 6688 , 998 , 4208 , 11184 , 835 , 317 , 1026 , 3337 , 842 , 6885 , 5607 , 5861 , 7157 , 6647 , 9140 , 831 , 5710 , 8878 , 836 , 5604 , 596 , 9776 , 3725 , 6714 , 2475 , 3303 , ATG5 , CYCS

Network weighting Automatically selected weighting method

Networks

A

Abu-Odeh-Aqeilan-2014 , Agrawal-Sedivy-2010 , Aichem-Groettrup-2012 , Albers-Koegl-2005 , Alexandru-Deshaies-2008 , Alizadeh-Staudt-2000 , Andresen-Flores-Morales-2014 , Arbuckle-Grant-2010 , Arroyo-Aloy-2014 , Arroyo-Aloy-2015

B

Bahr-Bowler-2013 , Bailey-Hieter-2015 , Bandyopadhyay-Ideker-2010 , Bantscheff-Drewes-2011 , Barr-Knapp-2009 , Barrios-Rodiles-Wrana-2005 , Behrends-Harper-2010 , Behzadnia-Lührmann-2007 , Bennett-Harper-2010 , Benzinger-Hermeking-2005 , Berggård-James-2006 , Bett-Hay-2013 , Bhatnagar-Attie-2014 , Bild-Nevins-2006 B , BIOGRID-SMALL-SCALE-STUDIES , BIOGRID-SMALL-SCALE-STUDIES , Blandin-Richard-2013 , Blomen-Brummelkamp-2015 , Blomen-Brummelkamp-2015 , Bogachek-Weigel-2014 , Boldrick-Relman-2002 , Bonacci-Soubeyran-2014 , Bouwmeester-Superti-Furga-2004 , Brajenovic-Drewes-2004 , Brehme-Superti-Furga-2009 , Bruderer-Hay-2011 , Burington-Shaughnessy-2008 , Butland-Hayden-2014 , Byron-Humphries-2012

C

Cai-Conaway-2007 , Camargo-Brandon-2007 , Campos-Reinberg-2015 , Cao-Chinnaiyan-2014 , Carmon-Liu-2014 , CELL_MAP , Chen-Brown-2002 , Chen-Ge-2013 , Chen-Huang-2014 , Chen-Zhang-2013 , Christianson-Kopito-2011 , Cloutier-Coulombe-2013 , Colland-Gauthier-2004 , Corominas-Iakoucheva-2014 , Couzens-Gingras-2013 , Cox-Rizzino-2013 , Coyaud-Raught-2015

D

Danielsen-Nielsen-2011 , Dart-Wells-2015 , de Hoog-Mann-2004 , Diner-Cristea-2015 , Dobbin-Giordano-2005 , Drissi-Boisvert-2015 , Dyer-Sobral-2010

E

Emanuele-Elledge-2011 , Emdal-Olsen-2015 , Ewing-Figeys-2007

F

Fenner-Prehn-2010 , Floyd-Pagliarini-2016 , Foerster-Ritter-2013 , Fogeron-Lange-2013 , Foster-Marshall-2013 , Freibaum-Taylor-2010

G

G

Gabriel-Baumgrass-2016 , Galligan-Howley-2015 , Gao-Reinberg-2012 , Gautier-Hall-2009 , Giannone-Liu-2010 , Glatter-Gstaiger-2009 , Gloeckner-Ueffing-2007 , Goehler-Wanker-2004 , Golebiowski-Hay-2009 , Goudreault-Gingras-2009 , Grant-2010 , Greco-Cristea-2011 , Grossmann-Stelzl-2015 , Guarani-Harper-2014 , Gupta-Pelletier-2015

H

Hanson-Clayton-2014 , Hauri-Gstaiger-2013 , Havrylov-Redowicz-2009 , Havugimana-Emili-2012 , Hayes-Urbé-2012 , Hegele-Stelzl-2012 A , Hegele-Stelzl-2012 B , Hein-Mann-2015 , Hill-Livingston-2014 , HUMANCYC , Humphries-Humphries-2009 , Hutchins-Peters-2010 , Huttlin-Gygi-2015

I

I2D-BIND-Fly2Human , I2D-BIND-Mouse2Human , I2D-BIND-Rat2Human , I2D-BIND-Worm2Human , I2D-BIND-Yeast2Human , I2D-BioGRID-Fly2Human , I2D-BioGRID-Mouse2Human , I2D-BioGRID-Rat2Human , I2D-BioGRID-Worm2Human , I2D-BioGRID-Yeast2Human , I2D-Chen-Pawson-2009-PiwiScreen-Mouse2Human , I2D-Formstecher-Daviet-2005-Embryo-Fly2Human , I2D-Giot-Rothbert-2003-Low-Fly2Human , I2D-INNATEDB-Mouse2Human , I2D-IntAct-Fly2Human , I2D-IntAct-Mouse2Human , I2D-IntAct-Rat2Human , I2D-IntAct-Worm2Human , I2D-IntAct-Yeast2Human , I2D-Krogan-Greenblatt-2006-Core-Yeast2Human , I2D-Krogan-Greenblatt-2006-NonCore-Yeast2Human , I2D-Li-Vidal-2004-CORE-1-Worm2Human , I2D-Li-Vidal-2004-non-core-Worm2Human , I2D-Manual-Mouse2Human , I2D-Manual-Rat2Human , I2D-MGI-Mouse2Human , I2D-MINT-Fly2Human , I2D-MINT-Mouse2Human , I2D-MINT-Rat2Human , I2D-MINT-Worm2Human , I2D-MINT-Yeast2Human , I2D-Ptacek-Snyder-2005-Yeast2Human , I2D-Tarassov-PCA-Yeast2Human , I2D-Tewari-Vidal-2004-TGFb-Worm2Human , I2D-vonMering-Bork-2002-High-Yeast2Human , I2D-vonMering-Bork-2002-Low-Yeast2Human , I2D-vonMering-Bork-2002-Medium-Yeast2Human , I2D-Wang-Orkin-2006-EScmplx-Mouse2Human , I2D-Wang-Orkin-2006-EScmplxlow-Mouse2Human , I2D-Yu-Vidal-2008-GoldStd-Yeast2Human , IMID , Ingham-Pawson-2005 , Innocenti-Brown-2011 , INTERPRO , IREF-BIND , IREF-BIOGRID , IREF-DIP , IREF-HPRD , IREF-INTACT , IREF-MATRIXDB , IREF-MPPI , IREF-PUBMED , IREF-SMALL-SCALE-STUDIES , IREF-SMALL-SCALE-STUDIES

J

Jeronimo-Coulombe-2007 , Jin-Pawson-2004 , Johnson-Kerner-Wichterle-2015 , Johnson-Shoemaker-2003 , Jones-MacBeath-2006 , Joshi-Cristea-2013 , Jäger-Krogan-2011

K

Kahle-Zoghbi-2011 , Kaltenbach-Hughes-2007 , Katsogiannou-Rocchi-2014 , Kim-Gygi-2011 , Kim-Major-2015 , Kneissl-Grummt-2003 , Koch-Hermeking-2007 ,

K

Kotlyar-Jurisica-2015 , Kristensen-Foster-2012 , Kärblane-Sarmiento-2015 , Kırlı-Görlich-2015

L

Lambert-Gingras-2015 , Lamoliatte-Thibault-2014 , Lau-Ronai-2012 , Lee-Songyang-2011 , Lehner-Sanderson-2004 A , Lehner-Sanderson-2004 B , Leng-Wang-2014 , Leung-Jones-2014 , Li-Chen-2015 , Li-Dorf-2011 A , Li-Dorf-2011 B , Li-Dorf-2014 , Li-Haura-2013 , Lim-Zoghbi-2006 , Lin-Smith-2010 , Lipp-Guthrie-2015 , Liu-Wang-2012 , Llères-Lamond-2010 , Loch-Strickler-2012 , Low-Heck-2014 , Lu-Zhang-2013 , Luo-Elledge-2009

M

Mak-Moffat-2010 , Mallon-McKay-2013 , Malovannaya-Qin-2010 , Markson-Sanderson-2009 , Maréchal-Zou-2014 , Matsumoto-Nakayama-2005 , McCracken-Blencowe-2005 , McFarland-Nussbaum-2008 , Meek-Piwnica-Worms-2004 , Milev-Mouland-2012 , Miyamoto-Sato-Yanagawa-2010 , Murakawa-Landthaler-2015

N

Nakayama-Ohara-2002 , Nakayasu-Adkins-2013 , Napolitano-Meroni-2011 , Narayan-Bennett-2012 , Nathan-Goldberg-2013 , NCI_NATURE , Neganova-Lako-2011 , Newman-Keating-2003 , Nicholson-Hupp-2014 , Noble-Diehl-2008

O

Oliviero-Cagney-2015 , Olma-Pintard-2009 , Oláh-Ovádi-2011 , Oshikawa-Nakayama-2012 , Ouyang-Gill-2009

P

Panigrahi-Pati-2012 , Papp-Lamia-2015 , Perez-Hernandez-Yáñez-Mó-2013 , Perou-Botstein-1999 , Perou-Botstein-2000 , Persaud-Rotin-2009 , Petschnigg-Stagljar-2014 , PFAM , Phillips-Corn-2013 , Pichlmair-Superti-Furga-2011 , Pichlmair-Superti-Furga-2012 , Pilot-Storck-Goillot-2010 , Povlsen-Choudhary-2012

R

Ramachandran-LaBaer-2004 , Raman-Harper-2015 , Ramaswamy-Golub-2001 , Ravasi-Hayashizaki-2010 , REACTOME , Reinke-Keating-2013 , Reyniers-Taymans-2014 , Richter-Chrzanowska-Lightowlers-2010 , Rieger-Chu-2004 , Rolland-Vidal-2014 , Rosenwald-Staudt-2001 , Roth-Zlotnik-2006 , Roux-Burke-2012 , Rowbotham-Mermoud-2011 , Roy-Pardo-2014 , Roy-Parent-2013 , Rual-Vidal-2005 A , Rual-Vidal-2005 B

S

Sang-Jackson-2011 , Sato-Conaway-2004 , Schadt-Shoemaker-2004 , Scholz-Taylor-2016 , Singh-Moore-2012 , Smirnov-Cheung-2009 , So-Colwill-2015 , Soler-López-Aloy-2011 , Sowa-Harper-2009 , Stehling-Lill-2012 , Stehling-Lill-2013 , Stelzl-Wanker-2005 , Stes-Gevaert-2014 , Stuart-Kim-2003 , Suter-Wanker-2013

T

Taipale-Lindquist-2012 , Taipale-Lindquist-2014 , Takahashi-Conaway-2011 , Tarallo-Weisz-2011 , Tatham-Hay-2011 , Teixeira-Gomes-2010 , Thalappilly-Dusetti-2008 , Thompson-Luchansky-2014 , Tong-Moran-2014 , Toyoshima-Grandori-2012 , Tsai-Cristea-2012

U

Udeshi-Carr-2012

V

van Wijk-Timmers-2009 , Vandamme-Angrand-2011 , Varjosalo-Gstaiger-2013 , Varjosalo-Superti-Furga-2013 , Venkatesan-Vidal-2009 , Vermeulen-Mann-2010 , Vinayagam-Wanker-2011 , Virok-Fülöp-2011 , Vizeacoumar-Moffat-2013

W

Wagner-Choudhary-2011 , Wallach-Kramer-2013 , Wan-Emili-2015 , Wang-Balch-2006 , Wang-Cheung-2015 , Wang-He-2008 , Wang-Maris-2006 , Wang-Xu-2015 , Wang-Yang-2011 , Weimann-Stelzl-2013 A , Weimann-Stelzl-2013 B , Weinmann-Meister-2009 , Wen-Wu-2014 , Whisenant-Salomon-2015 , Wilker-Yaffe-2007 , Willingham-Muchowski-2003 , Witt-Labeit-2008 , Wong-O'Bryan-2012 , Woods-Monteiro-2012 , Woodsmith-Sanderson-2012 , Wu-Garvey-2007 , Wu-Li-2007 , Wu-Ma-2012 , Wu-Stein-2010 , Wu-Stein-2010

X

Xiao-Lefkowitz-2007 , Xie-Cong-2013 , Xie-Green-2012 , Xu-Ye-2012

Y

Yang-Chen-2010 , Yatim-Benkirane-2012 , Yu-Chow-2013 , Yu-Vidal-2011

Z

Zanon-Pichler-2013 , Zhang-Shang-2006 , Zhang-Zou-2011 , Zhao-Krug-2005 , Zhao-Yang-2011 , Zhou-Conrads-2004 , Zhou-Hanemann-2016

Genes

Gene	Description	Rank
ATG5	autophagy related 5 [Source:HGNC Symbol;Acc:HGNC:589]	N/A
HSPA1A	heat shock protein family A (Hsp70) member 1A [Source:HGNC Symbol;Acc:HGNC:5232]	N/A
CAST	calpastatin [Source:HGNC Symbol;Acc:HGNC:1515]	N/A
ATG13	autophagy related 13 [Source:HGNC Symbol;Acc:HGNC:29091]	N/A
ATG12	autophagy related 12 [Source:HGNC Symbol;Acc:HGNC:588]	N/A
DNAJB1	DnaJ heat shock protein family (Hsp40) member B1 [Source:HGNC Symbol;Acc:HGNC:5270]	N/A
APAF1	apoptotic peptidase activating factor 1 [Source:HGNC Symbol;Acc:HGNC:576]	N/A
LAMP2	lysosomal associated membrane protein 2 [Source:HGNC Symbol;Acc:HGNC:6501]	N/A
CASP2	caspase 2 [Source:HGNC Symbol;Acc:HGNC:1503]	N/A
MAP2K5	mitogen-activated protein kinase kinase 5 [Source:HGNC Symbol;Acc:HGNC:6845]	N/A
CASP9	caspase 9 [Source:HGNC Symbol;Acc:HGNC:1511]	N/A
RAB1A	RAB1A, member RAS oncogene family [Source:HGNC Symbol;Acc:HGNC:9758]	N/A
MAP4K1	mitogen-activated protein kinase kinase kinase kinase 1 [Source:HGNC Symbol;Acc:HGNC:6863]	N/A
CYCS	cytochrome c, somatic [Source:HGNC Symbol;Acc:HGNC:19986]	N/A
SQSTM1	sequestosome 1 [Source:HGNC Symbol;Acc:HGNC:11280]	N/A
SPI1	Spi-1 proto-oncogene [Source:HGNC Symbol;Acc:HGNC:11241]	N/A
BID	BH3 interacting domain death agonist [Source:HGNC Symbol;Acc:HGNC:1050]	N/A
SOD1	superoxide dismutase 1, soluble [Source:HGNC Symbol;Acc:HGNC:11179]	N/A
MEF2C	myocyte enhancer factor 2C [Source:HGNC Symbol;Acc:HGNC:6996]	N/A
BCL2	B-cell CLL/lymphoma 2 [Source:HGNC Symbol;Acc:HGNC:990]	N/A
CASP8	caspase 8 [Source:HGNC Symbol;Acc:HGNC:1509]	N/A
CASP3	caspase 3 [Source:HGNC Symbol;Acc:HGNC:1504]	N/A
MTOR	mechanistic target of rapamycin [Source:HGNC Symbol;Acc:HGNC:3942]	N/A
MAP3K7	mitogen-activated protein kinase kinase kinase 7 [Source:HGNC Symbol;Acc:HGNC:6859]	N/A

Gene	Description	Rank
PSMD4	proteasome 26S subunit, non-ATPase 4 [Source:HGNC Symbol;Acc: HGNC:9561]	N/A
CDKN1A	cyclin dependent kinase inhibitor 1A [Source:HGNC Symbol;Acc:HGNC: 1784]	N/A
MAP2K1	mitogen-activated protein kinase kinase 1 [Source:HGNC Symbol;Acc: HGNC:6840]	N/A
JUN	Jun proto-oncogene, AP-1 transcription factor subunit [Source:HGNC Symbol;Acc:HGNC:6204]	N/A
SRC	SRC proto-oncogene, non-receptor tyrosine kinase [Source:HGNC Symbol; Acc:HGNC:11283]	N/A
CDC42	cell division cycle 42 [Source:HGNC Symbol;Acc:HGNC:1736]	N/A
TP53	tumor protein p53 [Source:HGNC Symbol;Acc:HGNC:11998]	N/A
BCL2L1	BCL2 like 1 [Source:HGNC Symbol;Acc:HGNC:992]	1
RPTOR	regulatory associated protein of MTOR complex 1 [Source:HGNC Symbol; Acc:HGNC:30287]	2
RAPH1	Ras association (RalGDS/AF-6) and pleckstrin homology domains 1 [Source:HGNC Symbol;Acc:HGNC:14436]	3
DIABLO	diablo IAP-binding mitochondrial protein [Source:HGNC Symbol;Acc: HGNC:21528]	4
PARD6B	par-6 family cell polarity regulator beta [Source:HGNC Symbol;Acc: HGNC:16245]	5
MAN1B1	mannosidase alpha class 1B member 1 [Source:HGNC Symbol;Acc:HGNC: 6823]	6
CRADD	CASP2 and RIPK1 domain containing adaptor with death domain [Source:HGNC Symbol;Acc:HGNC:2340]	7
FAS	Fas cell surface death receptor [Source:HGNC Symbol;Acc:HGNC:11920]	8
BRAF	B-Raf proto-oncogene, serine/threonine kinase [Source:HGNC Symbol; Acc:HGNC:1097]	9
CCS	copper chaperone for superoxide dismutase [Source:HGNC Symbol;Acc: HGNC:1613]	10
GRAP2	GRB2-related adaptor protein 2 [Source:HGNC Symbol;Acc:HGNC:4563]	11
ESR1	estrogen receptor 1 [Source:HGNC Symbol;Acc:HGNC:3467]	12
APPL1	adaptor protein, phosphotyrosine interacting with PH domain and leucine zipper 1 [Source:HGNC Symbol;Acc:HGNC:24035]	13
BCL2L11	BCL2 like 11 [Source:HGNC Symbol;Acc:HGNC:994]	14
BAD	BCL2 associated agonist of cell death [Source:HGNC Symbol;Acc:HGNC: 936]	15

Gene	Description	Rank
YWHAB	tyrosine 3-monooxygenase/tryptophan 5-monooxygenase activation protein beta [Source:HGNC Symbol;Acc:HGNC:12849]	16
BAK1	BCL2 antagonist/killer 1 [Source:HGNC Symbol;Acc:HGNC:949]	17
MAP3K3	mitogen-activated protein kinase kinase kinase 3 [Source:HGNC Symbol; Acc:HGNC:6855]	18
SOX18	SRY-box 18 [Source:HGNC Symbol;Acc:HGNC:11194]	19
TBP	TATA-box binding protein [Source:HGNC Symbol;Acc:HGNC:11588]	20
RNF223	ring finger protein 223 [Source:HGNC Symbol;Acc:HGNC:40020]	21
GSX1	GS homeobox 1 [Source:HGNC Symbol;Acc:HGNC:20374]	22
BAX	BCL2 associated X protein [Source:HGNC Symbol;Acc:HGNC:959]	23
PARD6G	par-6 family cell polarity regulator gamma [Source:HGNC Symbol;Acc: HGNC:16076]	24
XIAP	X-linked inhibitor of apoptosis [Source:HGNC Symbol;Acc:HGNC:592]	25
HSPA8	heat shock protein family A (Hsp70) member 8 [Source:HGNC Symbol; Acc:HGNC:5241]	26
TRAF6	TNF receptor associated factor 6 [Source:HGNC Symbol;Acc:HGNC: 12036]	27
NMT1	N-myristoyltransferase 1 [Source:HGNC Symbol;Acc:HGNC:7857]	28
MAPK7	mitogen-activated protein kinase 7 [Source:HGNC Symbol;Acc:HGNC: 6880]	29
BBC3	BCL2 binding component 3 [Source:HGNC Symbol;Acc:HGNC:17868]	30
TAB1	TGF-beta activated kinase 1/MAP3K7 binding protein 1 [Source:HGNC Symbol;Acc:HGNC:18157]	31
CAPN2	calpain 2 [Source:HGNC Symbol;Acc:HGNC:1479]	32
NOD1	nucleotide binding oligomerization domain containing 1 [Source:HGNC Symbol;Acc:HGNC:16390]	33
PDK1	pyruvate dehydrogenase kinase 1 [Source:HGNC Symbol;Acc:HGNC:8809]	34
BCAR1	BCAR1, Cas family scaffolding protein [Source:HGNC Symbol;Acc: HGNC:971]	35
RAF1	Raf-1 proto-oncogene, serine/threonine kinase [Source:HGNC Symbol;Acc: HGNC:9829]	36
FOS	Fos proto-oncogene, AP-1 transcription factor subunit [Source:HGNC Symbol;Acc:HGNC:3796]	37
RB1CC1	RB1 inducible coiled-coil 1 [Source:HGNC Symbol;Acc:HGNC:15574]	38
AVEN	apoptosis and caspase activation inhibitor [Source:HGNC Symbol;Acc: HGNC:13509]	39

Gene	Description	Rank
MAPK3	mitogen-activated protein kinase 3 [Source:HGNC Symbol;Acc:HGNC:6877]	40
ATG3	autophagy related 3 [Source:HGNC Symbol;Acc:HGNC:20962]	41
ATG16L1	autophagy related 16 like 1 [Source:HGNC Symbol;Acc:HGNC:21498]	42
RPL7	ribosomal protein L7 [Source:HGNC Symbol;Acc:HGNC:10363]	43
AL022067.1		44
PCNA	proliferating cell nuclear antigen [Source:HGNC Symbol;Acc:HGNC:8729]	45
RACK1	receptor for activated C kinase 1 [Source:HGNC Symbol;Acc:HGNC:4399]	46
FLOT1	flotillin 1 [Source:HGNC Symbol;Acc:HGNC:3757]	47
RHEB	Ras homolog enriched in brain [Source:HGNC Symbol;Acc:HGNC:10011]	48
CASP7	caspase 7 [Source:HGNC Symbol;Acc:HGNC:1508]	49
RICTOR	RPTOR independent companion of MTOR complex 2 [Source:HGNC Symbol;Acc:HGNC:28611]	50

Networks

Physical Interactions 33.99%

IREF-PUBMED 7.70%

Physical Interactions with 571 interactions from iRefIndex

Gloeckner-Ueffing-2007 6.96%

A novel tandem affinity purification strategy for the efficient isolation and characterisation of native protein complexes. Gloeckner et al (2007). *Proteomics*

Physical Interactions with 100 interactions from BioGRID

IREF-MPPI 4.71%

Physical Interactions with 382 interactions from iRefIndex

Wu-Li-2007 3.32%

Systematic identification of SH3 domain-mediated human protein-protein interactions by peptide array target screening. Wu et al (2007). *Proteomics*

Physical Interactions with 927 interactions from iRefIndex

IREF-BIND 3.14%

Physical Interactions with 3,659 interactions from iRefIndex

Arroyo-Aloy-2014 2.65%

Charting the molecular links between driver and susceptibility genes in colorectal cancer. Arroyo et al (2014). *Biochem Biophys Res Commun*

Physical Interactions with 598 interactions from iRefIndex

IREF-INTACT 1.75%

Physical Interactions with 56,297 interactions from iRefIndex

IREF-BIOGRID 1.67%

Physical Interactions with 155,470 interactions from iRefIndex

IREF-DIP 0.93%

Physical Interactions with 4,470 interactions from iRefIndex

IREF-HPRD 0.86%

Physical Interactions with 34,206 interactions from iRefIndex

Stelzl-Wanker-2005 0.30%

A human protein-protein interaction network: a resource for annotating the proteome. Stelzl et al (2005). *Cell*

Physical Interactions with 3,225 interactions from iRefIndex

Predicted 26.00%

I2D-IntAct-Worm2Human 10.03%

The IntAct molecular interaction database in 2010. Aranda et al (2010). *Nucleic Acids Res*

Predicted with 1,409 interactions from I2D

I2D-MINT-Rat2Human 4.44%

MINT: a Molecular INTeraction database. Zanzoni et al (2002). *FEBS Lett*

Predicted with 572 interactions from I2D

Predicted 26.00%

I2D-IntAct-Rat2Human 4.39%

The IntAct molecular interaction database in 2010. Aranda et al (2010). *Nucleic Acids Res*

Predicted with 1,052 interactions from I2D

I2D-IntAct-Mouse2Human 2.20%

The IntAct molecular interaction database in 2010. Aranda et al (2010). *Nucleic Acids Res*

Predicted with 3,427 interactions from I2D

I2D-MINT-Mouse2Human 2.17%

MINT: a Molecular INTeraction database. Zanzoni et al (2002). *FEBS Lett*

Predicted with 971 interactions from I2D

Wu-Stein-2010 2.11%

A human functional protein interaction network and its application to cancer data analysis. Wu et al (2010). *Genome Biol*

Predicted with 87,829 interactions from supplementary material

I2D-BIND-Mouse2Human 0.67%

BIND--a data specification for storing and describing biomolecular interactions, molecular complexes and pathways. Bader et al (2000). *Bioinformatics*

Predicted with 1,186 interactions from I2D

Co-expression 19.95%

Innocenti-Brown-2011 2.76%

Identification, replication, and functional fine-mapping of expression quantitative trait loci in primary human liver tissue. Innocenti et al (2011). *PLoS Genet*

Co-expression with 603,765 interactions from GEO

Rieger-Chu-2004 2.50%

Toxicity from radiation therapy associated with abnormal transcriptional responses to DNA damage. Rieger et al (2004). *Proc Natl Acad Sci U S A*

Co-expression with 259,974 interactions from GEO

Ramaswamy-Golub-2001 2.29%

Multiclass cancer diagnosis using tumor gene expression signatures. Ramaswamy et al (2001). *Proc Natl Acad Sci U S A*

Co-expression with 275,113 interactions from supplementary material

Bahr-Bowler-2013 2.23%

Peripheral blood mononuclear cell gene expression in chronic obstructive pulmonary disease. Bahr et al (2013). *Am J Respir Cell Mol Biol*

Co-expression with 274,949 interactions from GEO

Rosenwald-Staudt-2001 1.96%

Relation of gene expression phenotype to immunoglobulin mutation genotype in B cell chronic lymphocytic leukemia. Rosenwald et al (2001). *J Exp Med*

Co-expression with 114,694 interactions from supplementary material

Perou-Botstein-1999 1.79%

Distinctive gene expression patterns in human mammary epithelial cells and breast cancers. Perou et al (1999). *Proc Natl Acad Sci U S A*

Co-expression with 65,069 interactions from supplementary material

Noble-Diehl-2008 0.99%

Co-expression 19.95%

Noble-Diehl-2008

Regional variation in gene expression in the healthy colon is dysregulated in ulcerative colitis. Noble et al (2008). *Gut*

Co-expression with 661,539 interactions from GEO

Wu-Garvey-2007 0.95%

The effect of insulin on expression of genes and biochemical pathways in human skeletal muscle. Wu et al (2007). *Endocrine*

Co-expression with 267,109 interactions from GEO

Mallon-McKay-2013 0.91%

StemCellDB: the human pluripotent stem cell database at the National Institutes of Health. Mallon et al (2013). *Stem Cell Res*

Co-expression with 585,265 interactions from GEO

Perou-Botstein-2000 0.81%

Molecular portraits of human breast tumours. Perou et al (2000). *Nature*

Co-expression with 185,068 interactions from supplementary material

Wang-Maris-2006 0.71%

Integrative genomics identifies distinct molecular classes of neuroblastoma and shows that multiple genes are targeted by regional alterations in DNA copy number. Wang et al (2006). *Cancer Res*

Co-expression with 264,023 interactions from GEO

Alizadeh-Staudt-2000 0.59%

Distinct types of diffuse large B-cell lymphoma identified by gene expression profiling. Alizadeh et al (2000). *Nature*

Co-expression with 90,336 interactions from supplementary material

Chen-Brown-2002 0.47%

Gene expression patterns in human liver cancers. Chen et al (2002). *Mol Biol Cell*

Co-expression with 282,241 interactions from supplementary material

Boldrick-Relman-2002 0.39%

Stereotyped and specific gene expression programs in human innate immune responses to bacteria. Boldrick et al (2002). *Proc Natl Acad Sci U S A*

Co-expression with 111,707 interactions from supplementary material

Burington-Shaughnessy-2008 0.36%

Tumor cell gene expression changes following short-term in vivo exposure to single agent chemotherapeutics are related to survival in multiple myeloma. Burington et al (2008). *Clin Cancer Res*

Co-expression with 290,538 interactions from GEO

Wang-Cheung-2015 0.25%

Genetic variation in insulin-induced kinase signaling. Wang et al (2015). *Mol Syst Biol*

Co-expression with 411,047 interactions from GEO

Pathway 11.23%

REACTOME 6.15%

Pathway with 24,913 interactions from Pathway Commons

NCI_NATURE 5.09%

Pathway with 10,122 interactions from Pathway Commons

Genetic Interactions 4.26%

Genetic Interactions 4.26%

BIOGRID-SMALL-SCALE-STUDIES 3.87%

Genetic Interactions with 489 interactions from BioGRID

Lin-Smith-2010 0.39%

A genome-wide map of human genetic interactions inferred from radiation hybrid genotypes. Lin et al (2010). *Genome Res*

Genetic Interactions with 4,820,370 interactions from supplementary material

Co-localization 2.43%

Chen-Huang-2014 1.46%

Using an in situ proximity ligation assay to systematically profile endogenous protein-protein interactions in a pathway network. Chen et al (2014). *J Proteome Res*

Co-localization with 559 interactions from BioGRID

Johnson-Shoemaker-2003 0.98%

Genome-wide survey of human alternative pre-mRNA splicing with exon junction microarrays. Johnson et al (2003). *Science*

Co-localization with 426,332 interactions from GEO

Shared protein domains 2.13%

INTERPRO 2.13%

Shared protein domains with 608,863 interactions from InterPro
