# Supplementary material for: PI3K and Inhibitor of Apoptosis Proteins Modulate Gentamicin- Induced Hair Cell Death in the Zebrafish Lateral Line
Source: Front Cell Neurosci. 2017 Oct 18;11:326. doi: 10.3389/fncel.2017.00326 (PMC5651234; doi:10.3389/fncel.2017.00326)
Supplement: Supplementary file 4 [file Image_1.PDF]

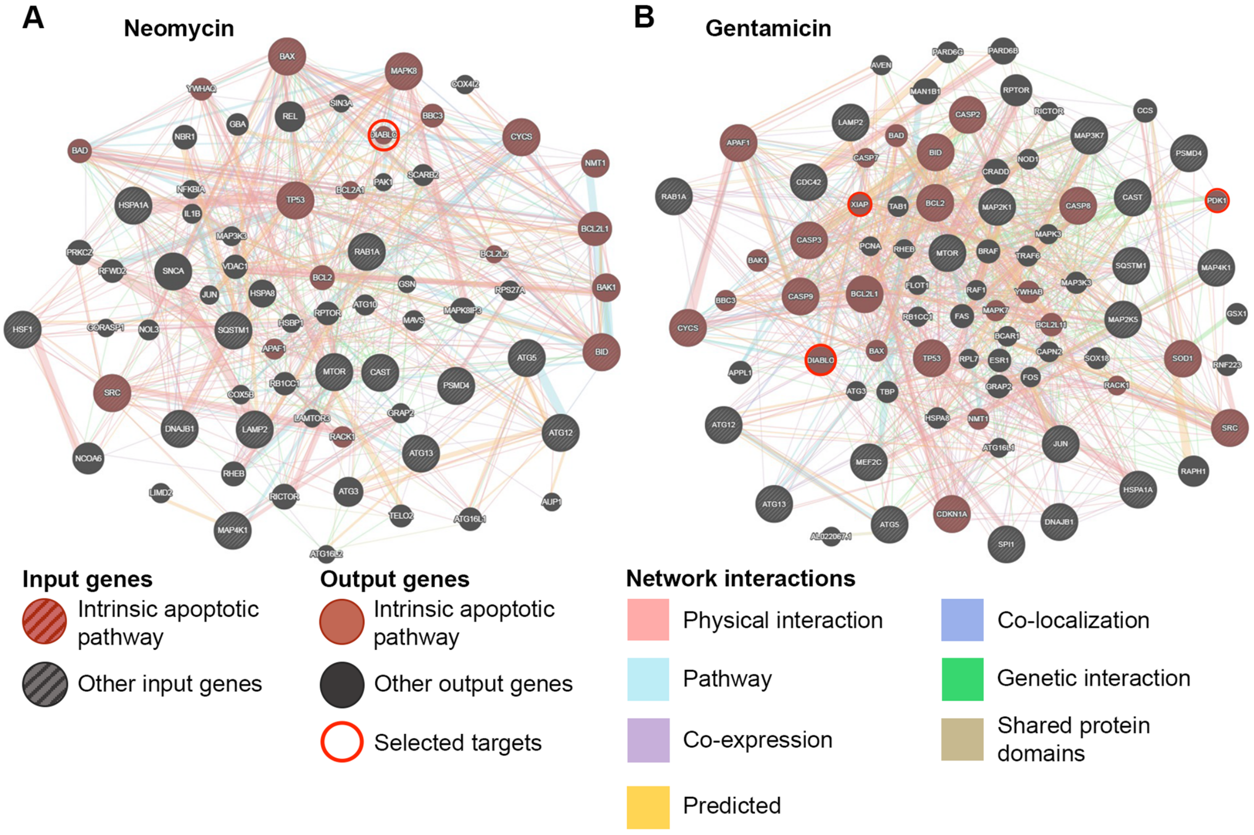

Supplemental Figure 1. Pathway analysis of neomycin- **(A)** and gentamicin- **(B)** induced hair cell death in the lateral line. Known molecular players from Coffin et al., (2013a, 2013b) were used as input (see Supplemental Table 1). Hatched circles are input genes for a given analysis. Red circles are known components of intrinsic apoptotic signaling pathways; those that are also input genes are hatched red. Dark gray or red circles that are not hatched are genes identified by the analysis as related to the input genes; these are candidate genes for future study. Red outlines indicate selected targets for the present study. Circle size is proportional to the number of interactions, based on GeneMANIA database analysis. Colored lines indicate different categories of network interactions, as indicated on the figure. Relationships between genes are often supported based on multiple types of data (*e.g.,* genetic interactions and co-localization data), which are displayed as multiple lines of different colors connecting two genes. More colored lines connecting two molecules indicate more evidence for relationships between these genes. The raw output of the GeneMANIA analysis is contained in Supplemental Files 2 and 3.
